# Comparative genomic analysis of the compound *Brassica napus Rf* locus

**DOI:** 10.1186/s12864-016-3117-0

**Published:** 2016-10-26

**Authors:** Lydiane Gaborieau, Gregory G. Brown

**Affiliations:** Department of Biology, McGill University, 1205 Doctor Penfield Ave., Montreal, QC H3A 1B1 Canada

**Keywords:** Cytoplasmic male sterility, Nuclear fertility restoration, Pentatricopeptide repeat, RFL gene, Retrotransposition, Nomadic gene

## Abstract

**Background:**

The plant trait of cytoplasmically-inherited male sterility (CMS) and its suppression by nuclear restorer-of-fertility (*Rf*) genes can be viewed as a genetic arms race between the mitochondrial and nuclear genomes. Most nuclear *Rf* genes have been shown to encode P-type pentatricopeptide repeat proteins (PPRs). Phylogenetic analysis of P-class PPRs from sequenced plants genomes has shown that Rf-proteins cluster in a distinct clade of P-class PPRs, RFL-PPRs, that display hallmarks of positive evolutionary selection. Genes encoding RFL-PPRs (*RFLs*) within a given plant genome tend to be closely related both in sequence and position, but a detailed understanding of how such species-specific expansion occurs is lacking. In the canola, (oilseed rape) species *Brassica napus*, previous work has indicated the nuclear restorer genes for the two native forms of CMS, *Rfn* (for *nap* CMS) and *Rfp* (*pol* CMS), represent alternate haplotypes, or alleles, of a single nuclear locus.

**Results:**

Fine genetic mapping indicates that *Rfn* does indeed localize to the same genomic region as *Rfp*. We find this region is enriched in *RFL* genes, three of which, based on their position and expression, represent potential candidates for *Rfn*; one of these genes, designated *PPR4*, is a preferred candidate in that it is not expressed in the *nap* CMS line. Comparison of the corresponding regions of the genomes of *B. rapa*, *B. oleracea*, *Arabidopsis thaliana* and *A. lyrata* provides insight into the expansion of this group of *RFL* genes in different lines of evolutionary descent.

**Conclusions:**

Unlike other nuclear restorer loci containing multiple *RFL* genes, the *RFL* genes in the *Rf* region of *B. napus* are not present in tandem arrays but rather are dispersed in genomic location. The genes do not share similar flanking non-coding regions and do not contain introns, indicating that they have duplicated primarily through a retrotransposition-mediated process. In contrast, segmental duplication has been responsible for the distribution of the 10 sequences we annotated as *RFL* genes in the corresponding region of the *A. lyrata* genome. Our observations define the Brassica *Rf* locus and indicate that different mechanisms may be responsible for the proliferation of *RFL* genes even among closely related genomes.

**Electronic supplementary material:**

The online version of this article (doi:10.1186/s12864-016-3117-0) contains supplementary material, which is available to authorized users.

## Background

Cytoplasmic male sterility (CMS) is a widespread, maternally inherited trait of flowering plants that results from the expression of novel genes in the mitochondrial genome. The novel genes are unique for each type of CMS and often are chimeric in structure, consisting of segments of standard mitochondrial genes fused, in frame, to other open reading frames that bear no resemblance to known functional genes [1]. The novel gene products associated with CMS are inner membrane proteins whose presence is thought to compromise mitochondrial function, although the specific mechanisms whereby this leads to an abrogation of functional pollen formation, are, with the exception of a few examples (e.g. [2–4]), not clear.

The CMS phenotype is often masked by the presence of nuclear restorer genes. These genes are specific to each form of CMS and in general act to down-regulate, at the post-transcriptional level, the expression of the cognate novel CMS-causing mitochondrial genes. Consequently CMS can often only be revealed through wide intraspecific or interspecific crosses. CMS is also revealed in nature via gynodioecy, populations consisting of a mixture of female (i.e. male sterile) and hermaphroditic plants. Theoretical studies have indicated that maternally inherited male sterility can, under certain circumstances, spread in populations; if the frequency of male sterile individuals then becomes sufficiently high, pollen becomes scarce, a condition favoring the counter-selection of nuclear restorers that suppress the male-sterility [5–7]. This type of interaction can be viewed in the context of genomic conflict: the selective interests of the uniparentally inherited mitochondrial genome oppose those of the biparentally-inherited nuclear genome [6]. Such genomic conflicts are often characterized as a genetic arms race: the appearance of a new male-sterility mitochondrial gene will drive the appearance of a corresponding restorer gene, analogous to the “gene-for-gene” selection of new host resistance genes in response to new pathogen races [8, 9].

A number of nuclear restorers have been isolated in recent years [10–18]. Most of these have been shown to encode proteins composed of tandem repeats of a degenerate 35 amino acid sequence, the pentatricopeptide repeat (PPR) [19]. Most eukaryotic genomes harbor only a few PPR-encoding genes, but in plants this gene family has greatly expanded, and contains plant specific forms with repeats that are longer and shorter than the canonical 35 amino acid motif [20]. Most plant PPR proteins are targeted to the mitochondria and chloroplasts, and bind to specific RNA substrates, mediating numerous aspects of post-translational gene expression including splicing, nuclease processing, editing and translation [21]. Restorer PPR proteins are largely composed exclusively of core repeat motifs as defined by the P-type PPR subfamily, and comprise a distinct phylogenetic clade of such proteins, Rf-like PPRs, whose corresponding coding sequences are designated as *RFL* genes [22]. Comparison of the *RFL* genes within a given species reveals evidence of positive evolutionary selection, consistent with the premise of genomic conflict [22–24]. Moreover, the position of certain *Rf-like PPR* genes are not conserved between otherwise closely-related, syntenic chromosomal regions [23] giving these genes a “nomadic”-like character.

Two native CMS systems, *nap* and *pol*, are known to occur in the oilseed rape (canola) species *Brassica napus*. The causative factor for the “Polima” or *pol* CMS, *orf224,* is a novel CMS-associated mitochondrial gene in which the promoter and first 58 codons of the *atp8* gene are fused to a unique sequence bearing little similarity to other known sequences [25]. *nap* CMS is specified by *orf222*, a chimeric gene that is similar but not identical over its entire length to *orf224* [26]. The two genes are located in different positions on the mitochondrial genome: *orf224* is situated upstream of the *atp6* gene, while *orf222* is located upstream of *nad5* exon c*.* The mitochondrial genome of the male fertile *B.napus* cytoplasm, *cam*, is identical to that found in one of the progenitor species, *B. rapa* (formerly *campestris,* see below) and lacks both *orf222* and *orf224*. The restorers for the *pol* and *nap* systems, *Rfp* and *Rfn*, respectively, each down-regulate the expression of their cognate CMS-associated mitochondrial genes by mediating RNA cleavage events within unique regions of the corresponding transcripts [25, 26]. The *Rfn* allele is also associated with additional RNA cleavage events in the coding regions of *nad4* and *ccmF*_*N2*_ (*formerly ccl1-l* [27, 28]) which are not observed in plants homozygous for the *Rfp* allele or for the non restoring, or universal maintainer genotype *rf* [29]. Indeed, *Rfn* and *Rfp* represent distinct alleles or haplotypes of a single nuclear locus, and it has not been possible to separate these genes or their associated mtRNA cleavage properties via genetic crosses involving the three nuclear and cytoplasmic genotypes [8]. Together, these characteristics indicate that two CMS-restorer systems have evolved in the relatively recent evolutionary past, and that *B. napus* CMS systems therefore offer an attractive model for understanding the molecular events underlying the evolution of CMS-restorer gene systems [29].

Further progress towards this goal requires the identification and characterization of *Rfp* and *Rfn* genes and an understanding of the evolution of the genomic regions surrounding them. The complexity of the amphidiploid *B. napus* genome has posed a key challenge in the accomplishment of this goal. Following the evolutionary divergence of the Brassica and Arabidopsis lineages about 35 Mya [30, 31] polyploidization events gave rise to an ancestral Brassica genome in which most *Arabidopsis thaliana* regions were present as 3 co-linear copies [32]. Modern diploid Brassica genomes reflect further genomic rearrangement, gene loss and gene duplication and show fragmented co-linearity with Arabidopsis, with each co-linear region being represented, on average, in three copies. *B. napus* is the product of a relatively recent interspecific hybridization between two such Brassica species, *B. rapa* (source of the A genome) and *B. oleracea* (source of the C genome), which diverged from one another between 2.6 and 4.2 Mya [33].

We have previously introgressed the *Rfp* gene into the *B. rapa* genome and have fine-mapped the gene to a segment of the *B. napus* genome co-linear with *A. thaliana* chromosome 1 coordinates 4.28–4.40 [34, 35]. We report here our genetic localization of the *Rfn* gene and the molecular characterization of the genome regions surrounding it. We identify a preferred candidate for *Rfn*. In addition, by analyzing the positions and sequence relationships between the *RFL* genes in related regions of Arabidopsis and Brassica genomes, we are able to draw inferences regarding the molecular events through which this gene family has expanded and the *Rf* locus evolved.

## Results

### Identification of a BAC clone corresponding to the *Rfp/Rfn* region

**Fig. 1:**
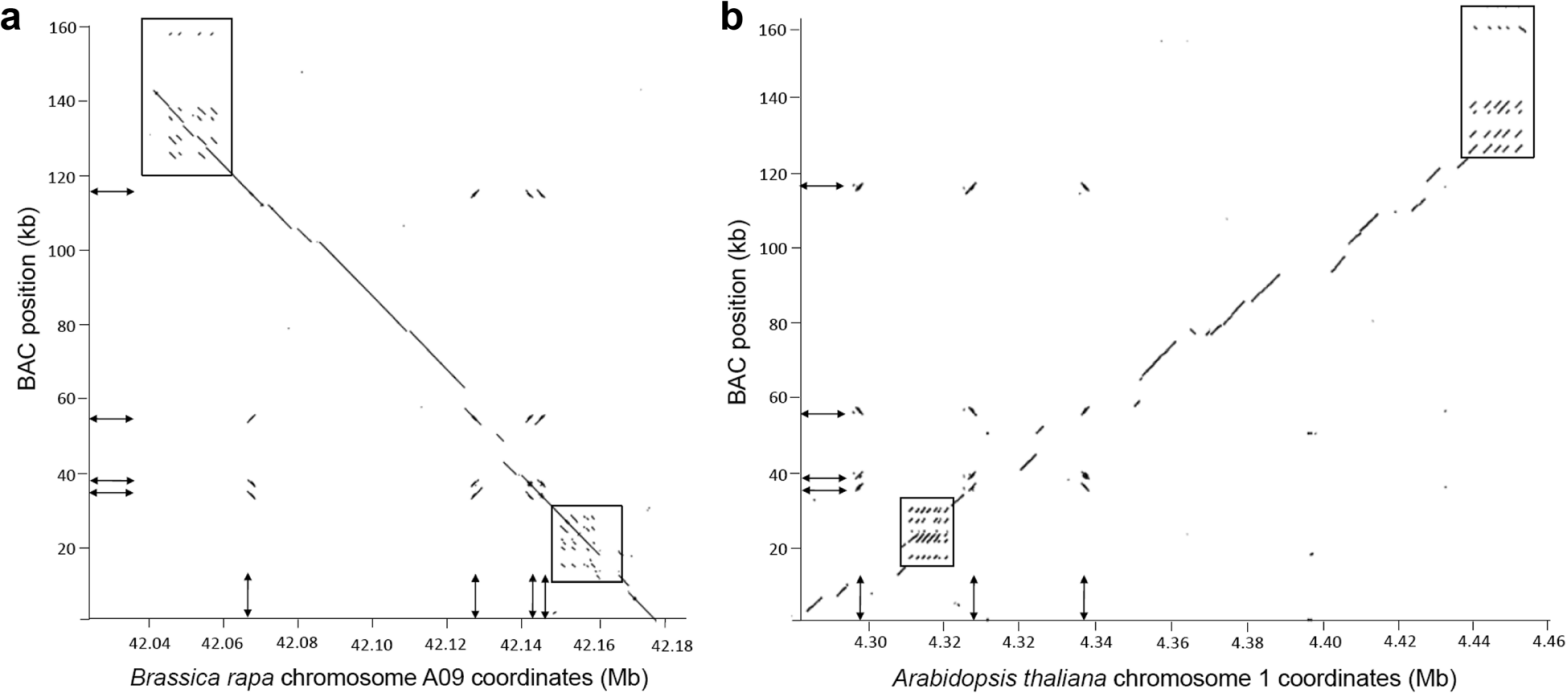
Dot matrix alignments of the sequence of a bacterial artificial chromosome (BAC) anchored in the *Rfp* region with the syntenic regions of *B. rapa* chromosome A09 (**a**) and *A. thaliana* chromosome 1 (**b**). Boxed regions indicate regions encoding short repeated gene/pseudogene sequences. The arrows indicate repeated regions showing similarity to four and three distinct sites in *B. rapa* and *A. thaliana* respectively

A *B. rapa* cosmid clone containing a polymorphism tightly linked to *Rfp*, a SNP in the gene corresponding to *A. thaliana* At1g12910 at chromosome 1 coordinate 4.395 [35], was recovered, sequenced and used as a source of probes to screen a *B. napus* bacterial artificial chromosome (BAC) library derived from a line with the *Rfn* genotype (see Methods) at the restorer locus. A single BAC, of approximately 180 kb, designated NO202E11, containing a sequence identical to the corresponding region of the cosmid probe was selected and sequenced. Sequence analysis showed that the BAC contained a sequence highly similar to the At1g12910 gene with the *Rfn*-linked allele of the Atg12910-orthologous SNP. Since our previous studies indicated *Rfp* and *Rfn* are different haplotypes of the same genomic region, we considered this BAC to likely be anchored in the *Rfn* region. The BAC sequence was found to be collinear with the region of the *A. thaliana* genome extending from chromosome 1 coordinates 4.27–4.47 and with the region of the *B. rapa* genome extending from chromosome A09 coordinates 42.18 Mb to 41.79 Mb. Dot matrix visualization of the synteny between the sequenced BAC and the *B. rapa* and *A. thaliana* genomic regions is shown in Figs. 1a and 1b, respectively.

### High resolution mapping of the *Rfn* gene

The premise that *Rfp* and *Rfn* are alleles or closely linked alternative haplotypes of the same genetic locus is based on relatively rough genetic mapping and genetic crosses in which the transcript modification activities associated with the two genes have been found to be mutually exclusive [8]. We would therefore expect that higher resolution genetic mapping studies should confirm that *Rfn* does, indeed, co-localize with the region in the selected BAC. We constructed a BC1 mapping population, genotyped individuals with SNPs from the region of *B. napus* chromosome A09 corresponding to *B. rapa* coordinates 39.75 Mb to 43.27 Mb. One of these SNPs, localized at coordinate 42.13 Mb, fell within the BAC sequence. Specific information on the SNPs that were polymorphic between the mapping parents of the cross is provided in Additional file 1: Table S1.

**Fig. 2:**
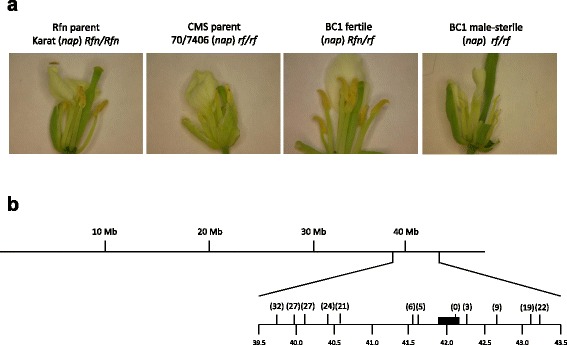
Mapping of the *Rfn* gene on chromosome A09. **a** Flowers from the parents and BC1 progeny of the mapping cross. Petals have been removed to allow display of anther morphology. Cytoplasm is designated in parentheses. The male parent of the cross and fertile progeny heterozygous for *Rfn* (*Rfn/rf*) have anthers with normal morphology and shed abundant pollen. The male sterile (CMS) parent and BC1 progeny homozygous for the recessive maintainer allele (*rf/rf*) have stamens with short filaments and underdeveloped anthers that shed little or no pollen. **b** Location of *Rfn* on chromosome A09. The region of the *B. rapa* chromosome chosen as range for targeted mapping extending from coordinates 39.5 to 43.5 Mb is expanded to illustrate the mapping results. Numbers in parentheses indicate the number observed recombination events between a marker at that location and the *Rfn* gene. The filled rectangle indicates position of the BAC spanning the region delimited in *Rfp* mapping experiments. No recombination was observed between the single marker located within the BAC and the *Rfn* gene

The *nap* CMS phenotype is leaky in a temperature-dependent, cultivar-specific manner [36, 37], a challenge for the precise mapping of *Rfn.* The choice of the *Rfn* parent for the mapping cross is therefore critical. Based on our previous work, we knew that utilizing the cultivar “Karat” could provide a BC1 mapping population that would allow us to satisfactorily distinguish between CMS and fertility restored progeny [8]. To derive our BC1 population, two F1 individuals generated by pollinating CMS plants (*rf/rf* [*nap*]) with Karat (*Rfn/Rfn* [*nap*]) were crossed back as females to the Karat parent. Of 293 individual BC1 plants, 146 were unambiguously scored as fertile (*Rfn/rf* [*nap*]) and 147 as male sterile (*rf/rf* [*nap*]). The floral phenotypes of the parental and fertile and sterile progeny are illustrated in Fig. 2a. Genotyping of the population, as illustrated in Fig. 2b, allowed us to map the *Rfn* gene to the region of *B. napus* chromosome BnA09 containing the selected BAC, confirming that *Rfp* and *Rfn* were closely linked alleles. The single marker positioned within the BAC was perfectly linked to *Rfn*. Because both *Rfp* and *Rfn* are located within this chromosomal region we henceforth refer to it as the *B. napus Rf* locus.

To more precisely localize *Rfn* we identified additional polymorphic molecular markers mapping within the region delimited by the SNPs. Primers designed to amplify genomic regions extending across introns in genes located between the SNPs most proximal to *Rfn* identified two intron length polymorphisms (ILPs, [38]), amplification products that differed in size between the two parents of the mapping population. When no noticeable amplicon length difference was evident, the products were further subjected to restriction cleavage to reveal cleaved amplified polymorphisms (CAPS, [39]). This strategy allowed four additional polymorphic markers anchored in the targeted region to be identified. These markers were then used to genotype individuals in which recombination had occurred between the closest flanking SNPs. This strategy allowed us to delimit the *Rfn* containing region to the segment of corresponding *B. rapa* chromosome A09 coordinates 41.68–42.58 Mb.

### Characteristics of the *B. napus Rf* locus

**Fig. 3:**
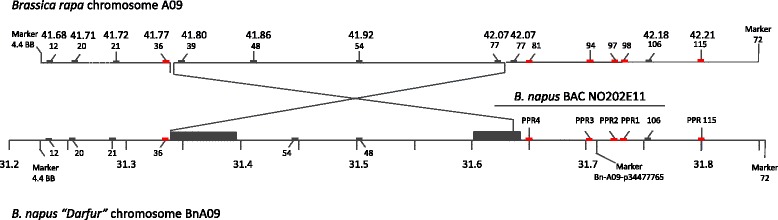
Organizational differences in the Brassica *Rf* locus between *B. rapa* and *B. napus*. Locations of specific genes appear as thick bars on the lines representing the two genomes. Genes are indicated to according to the Phytozome *B. rapa* designations (i.e. 12 corresponds to Brara.I05012). Map coordinates in Mb appear at specific intervals in the representation of the *B. napus* genome (*lower bar*), and above the coordinates of the corresponding genes in the *B. rapa* genome representation (*upper bar*). *RFL* genes are designated as red bars. The dotted line between genes 20 and 21 of the *B. rapa* genome is used to indicate the absence of genes that are present at this site in the *B. napus* genome. The crossed lines between the genomes indicate that site of a major sequence inversion. The filled boxes on the *B. napus* genome indicate are used to indicate the regions containing duplicated F-box and Cytochrome P_450_ CypA encoding genes that may have been involved in the sequence rearrangement. Markers 4.4BB and 72 genetically delimit the region that can potentially encode *Rfn*. Bn-A09-p34477765 is a SNP that shows complete genetic linkage to *Rfn*

Several interesting features were revealed through dot matrix visualization of the synteny between the sequenced BAC and the *B. rapa* and *A. thaliana* genomic regions (Figs. 1a and 1b, respectively). Regions at each end of the BAC, roughly located at positions 10–30 and 120–140 kb, showed similarity to sets of short repeated sequences, shown as boxed regions on the figures. Detailed comparative annotation of the genes in the BAC with the corresponding portions of the *B. rapa* and *A thaliana* (Additional file 2: Table S2) indicated that the repeated sequences corresponded to sets of directly repeated genes and/or pseudogenes encoding thionin (PR-13) proteins (positions 10–13 kb) in one case and Cytochrome P_450_ Cyp2 proteins (positions 120–140 kb) in the other. Comparative annotation with the more recently released *B. napus* cv. “Darfur” chromosome BnA09 [40] indicated that a sequence inversion has taken place in *B. napus* by which the sequence extending from *B. rapa* chromosome A09 coordinates 41.80–42.04 Mb is inverted (Additional file 3: Table S3). The inverted region is flanked by sequence spans encoding Cytochrome P_450_ Cyp2 and F-box domain encoding genes. This rearrangement appears as a gap in synteny from BAC coordinates 120–140 kb in both the *B. rapa* and *A, thaliana* plots. At around coordinate 160 kb, near the very end of the BAC, sequence similarity is observed between the BAC and the Cytochrome P_450_ Cyp2 genes, but in reverse orientation, as expected in the case of an inversion. Key features of the differences between the BAC, *B. rapa* and *B. napus* cv. “Darfur” genomes are illustrated in Fig. 3.

### Rf-like PPR genes in the *B. napus* Rf locus

Another striking feature of the dot matrix comparisons was the presence of sequences located near BAC coordinates 34, 38, 52 and 118 kb (illustrated by double headed arrows in Figs. 1a and 1b) that mapped to four corresponding sites in *B. rapa* chromosome A09. In the Arabidopsis genome, only three of the four sites were located at corresponding positions; no Brassica sequence was found to correspond to the site located near the 4.295 Mb coordinate on Arabidopsis chromosome 1. Inspection of the Brassica repeat sequences indicated that they all corresponded to regions encoding highly similar *RFL* PPR genes predicted to be targeted to the mitochondria, which we designated as *PPR1-4*.

Because the genetically defined limits of the *Rfn* region extended beyond the boundaries of the BAC, we searched the corresponding region of the *B. rapa* chromosome 9 for additional *RFL* PPRs that could serve as candidates for *Rfn*. We were able to identify two more such genes, one B.rara.I05036, located between coordinates 41.777 and 41.776 Mb, and the other, B.rara.I05115, between coordinates 42.211 and 42.213 Mb. Orthologous PPR genes at corresponding locations were found to be present on *B. napus* “Darfur” chromosome BnA09 and are designated *Bn036* (coordinates 31.334–31.341 Mb) and *Bn115* (31.797–31.806 Mb). Both of these genes encode products predicted to be targeted to the mitochondrion. Notably, *Bn036* is located roughly 100 kb from the flanking marker 4.4BB but 300 kb from the *RFL* genes in the BAC. No genes encoding PPR proteins other than *RFL* genes were detected in the region. A preliminary phylogenetic analysis (Additional file 4: Figure S1) indicated that five of the six genes clustered with three *A. thaliana RFL* genes located on the long arm of chromosome 1, *AtRFL2* (At1g12300), *AtRFL3* (At1g12620) and At1g12775 (re-annotated At1g12770 or *AtRFL25*). The sixth gene *PPR2*, clustered within a neighboring branch with its ortholog *AtRFL4* (At1g12700). *PPR3* and *AtRFL25*, like *PPR2* and *AtRFL4,* are located at matching positions in their corresponding chromosomes.

**Fig. 4:**
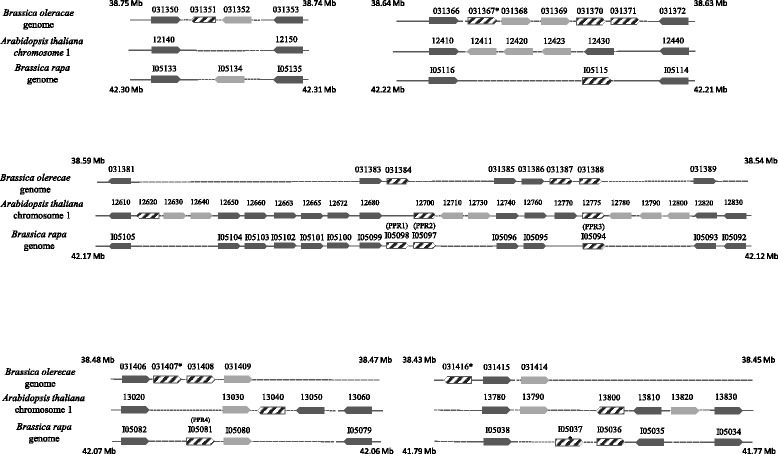
Position of *RFL* and surrounding genes in the *B. napus/rapa Rf* locus andrelated genome regions. Genes are indicated by wide arrows, with the direction of the arrows indicating the 5’ to 3’ orientation of the coding sequence. *RFL-PPR* genes are hatched. Dark filling indicates genes conserved in location among the three genomes, light filling a gene that is absent at the same position in one or both of the other genomes. Numbers reflect the online gene annotation as prefixed by Bol, At1g and Brara, in *B. oleracea, A. thaliana* and *B. rapa*, respectively. Asterisks (*) indicate *RFL* genes considered too small to be included in the Phylogeny of Fig. 7. The position of the different displayed segments of the *B. oleracea* and *B. rapa* genomes are indicated by the coordinates flanking each segment of the corresponding chromosomes (C08 and A09) from the BRAD (http://brassicadb.org/brad/) online resource

A comparison of the relative positions of the genes in the *A. thaliana* and *B. rapa/B. napus* A genomes is presented in Fig. 4. The phylogenetic analysis indicated that all of the Brassica genes represented Rf-like PPRs, as did three of the four Arabidopsis genes; a non-*RFL A. thaliana* PPR gene, At1g13030, is found at a site close to but not precisely matching the Brassica *RFL PPR4* gene at coordinate 118 kb in the BAC. One of the genes, At1g12700 (*AtRFL4*, [41]) located at a matching position in both genomes, is known to encode a mtRNA processing factor, RPF1, which confers nuclease cleavage events on *nad4* transcripts, which are also a target of the Brassica *Rfn* gene. These observations revealed six *RFL B. napus* PPR genes that could serve as candidates for *Rfn*. One of the genes, *PPR4*, has been previously proposed as a candidate for *Rfp* on the basis of fine mapping data [42]. None of the Brassica PPR genes were predicted to contain an intron, an observation confirmed by RT-PCR analysis of floral transcripts (see below).

### Expression of the *Rf*-region *RFL* genes in *nap* CMS and fertility restored plants

**Fig. 5:**
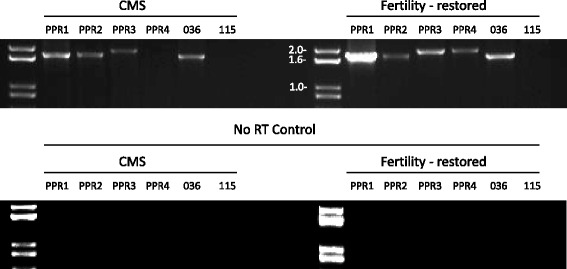
Expression of *Rf* locus *B. napus PPR* genes. Total RNA was extracted from the CMS and restorer parents of the mapping cross and analyzed by RT-PCR. The lower panel shows the results of the experimental control, performed at the same time on the same group of samples, in which reverse transcriptase was omitted prior to PCR amplification of cDNA products

The observation that *rf-PPR592,* the non-restoring allele of the petunia restorer, *Rf-PPR592,* is not expressed but is otherwise similar to the restorer suggested that expression differences among different candidate PPR genes could be used as a tool to prioritize candidates for further analysis of restoration function. We used RT-PCR to examine the expression of the six candidate *RFL* genes located within the Brassica *Rf*-locus. As shown in Fig. 5, we did not detect expression of *Bn115* in floral buds of either CMS or nuclear restored plants. Of the remaining 5 *B. napus* Rf-region *RFL* genes, expression of one, *PPR4,* was detected in the buds of nuclear fertility restored but not CMS plants; *PPR4*, is thus seen a strong candidate for *Rfn. PPR1* and *PPR3* both also show higher levels of expression in restored than in CMS flowers.

The sequences of the RT-PCR products, as shown in Additional file 5: Figure S2, provided further information relevant to the structures of the genes and their possible roles in nuclear fertility restoration. The sequences of the transcripts were co-linear with the corresponding genomic DNA sequence, indicating that, as expected, these genes lacked introns. Interestingly, a termination codon was found at nucleotide position 1119 in the coding sequence of the RT-PCR products of *PPR2* from both the CMS and nuclear restored lines. This termination codon was not detected in genomic sequences of either *B. rapa* or the BAC NO202E11, and resulted from a one nucleotide insertion in the CMS and restorer sequences prior to the termination codon, followed by a two nucleotide insertion in the same sequences.

### Expansion of a family of Rf-like PPR genes within Brassica genomes

The *B. napus* genome is derived from a recent interspecific hybridization event between the C genome species *B. oleracea* and the A genome of *B. rapa,* two species which diverged in descent approximately 3.7 Mya*.* Because *RFL* PPR genes are known to be variable in chromosomal position between closely related genomes [22, 23], it was of interest to determine the position of the *Rfn* candidates and close paralogs in the *B. oleracea* C genome. To accomplish this we first identified close homologs of the different *B. napus* Rf region PPR genes on the *B. oleracea* genome using the blastn resource of the Brassica database (BRAD, [43]). We found a cluster of highly similar sequences between chromosome 8 coordinates 38.54 and 38.75 Mb, a region over which synteny was maintained with the corresponding regions *of A. thaliana* and *B. rapa*. The annotation of the region indicated that the homologous sequences corresponded to 10 highly related *RFL*-PPR genes. Eight of these genes lacked predicted introns, whereas two, Bol31351 and Bol31388, were each predicted to contain a single intron.

The relative position of PPR genes in the *A. thaliana*, *B. rapa/napus* (A) and *B. oleracea* (C) genomes in the region over which synteny is conserved among the three genomes is illustrated in Fig. 4. Seven of the 10 C genome *B. oleracea* PPR genes are located a position corresponding their location in the A genome. In three cases, involving Bol31370/Bol31371, Bol31387/Bol31388 and Bol313407/Bol313408, a tandem pair of *B. oleracea* PPR genes is found at sites occupied by only a single PPR gene in the A genome. In one case a single *B. oleracea* gene, Bol31384, was found at a site containing two adjacent PPRs in the A genome. These observations indicated that the relative locations of the majority of Rf region PPR genes have not changed since the A/C genome divergence.

### Positional variation of *RFL* genes in the Rf-orthologous regions of two Arabidopsis genomes

Because we observed some conservation of location between some *A. thaliana RFL* genes and Brassica *RFL* genes in the Rf-region, it was of interest to determine to what extent this positional conservation could also be observed between the *A.thaliana* and *A. lyrata* genomes, which diverged from each other between 4 and 5 Mya. Fujii et al. [22] observed the *RFL* genes in the *A. thaliana* region orthologous to the Brassica Rf-region (Fig. 1a) fell into *RFL* subgroup 1, most of which are located between the 4.18 and 4.33 Mb coordinates of chromosome 1. *A. lyrata* subgroup 1 genes similarly cluster within a 221 kb segment of the scaffold 1 genome assembly unit.

**Fig. 6:**
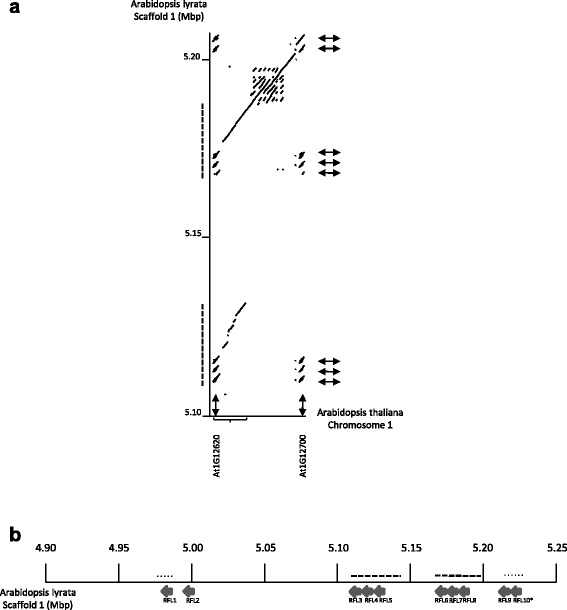
*RFL* genes in a region of the *A. lyrata* genome orthologous to the region of the *A. thaliana* genome depicted in Figs. 1 and 3. **a** A dot matrix representation illustrating the duplication of a region containing 3 *RFL* genes at positions indicated by the double headed in the *A. lyrata* genome; all three genes and the surrounding sequences correspond to the *AtRFL3* (At1g12620) region of *A. thaliana.* The bracket indicates the region of the Arabidopsis genome that is duplicated in *A. lyrata* (dotted lines along the y axis). A second duplication occurs at the position of *AtRFL4* (At1g12700); in this case the duplication involves the *AtRFL2* gene (At1g12300). **b** Depiction of the analyzed segment of the *A. lyrata* genome. Arrows indicate the direction and orientation of the RFL genes. The dotted lines indicate duplicated segments: one containing three genes at the location corresponding to *AtRFL3* and the other containing two genes apparently derived from *AtRFL2*, with one copy at the location of *AtRFL2* and the other at the location of *AtRFL4*

Dot matrix visualization of the similarities between these two Arabidopsis genomic regions revealed several interesting features of the positional relationships among these genes (Fig. 6) At the position corresponding to *AtRFL2* (At1g12300), two highly similar genes (indicated by the double-headed arrows in Fig. 6a), designated in Fig. 6b as *AlyRFL1* and *AlyRFL2* 1 *,* are found at the corresponding site in the *A. lyrata* genome. The 5’ and 3’ non-coding regions surrounding these genes are similar to one another, indicating that the two *lyrata* genes arose from a tandem duplication of a region encoding an *AtRFL2*-like gene. At the position corresponding to *AtRFL3* (At1g12620), a tandem triplication of a similar gene was found at two different positions in the *A. lyrata* genome. This arrangement arose from the duplication of an approximately 14 kb region spanning the triplication and extending in the direction matching the centromere proximal side of *A. thaliana* chromosome 1 (Fig. 6a). Thus six *RFL* genes, which we designate as *AlyRFL3-9*, are found within a duplication spanning the region around *AtRFL3*. Another tandem duplication with sequence similarity to the *AlyRFL1/AlyRFL2* pair is found at a site corresponding to *AtRFL4* (At1g12700). These observations suggest segmental duplication is the primary mechanism behind the proliferation of this family of *RFL* genes in this region the *A. lyrata* genome.

### Phylogenetic relationships among Brassica and Arabidopsis RFL proteins

**Fig. 7:**
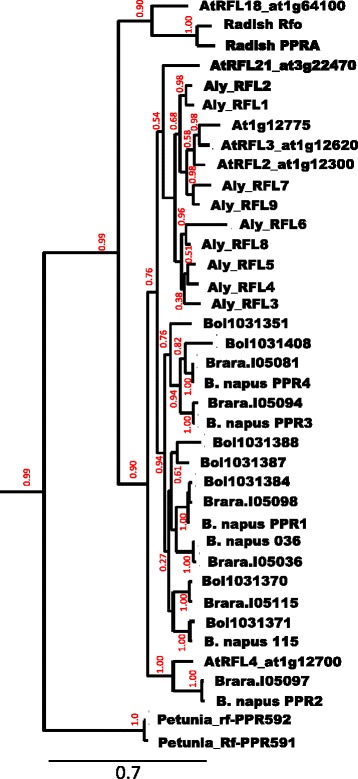
Phylogeny of Brassica and Arabidopsis PPR proteins. A maximum likelihood tree was generated by the PhyML resource using as input the sequences of known dicot restorer proteins and related orthologs, the predicted protein sequences of *A. thaliana* subgroup 1 RFL proteins, *A. lyrata* proteins predicted from our analysis of the targeted region enriched in subgroup 1 RFL proteins [22], and the *B. napus*, *B. rapa* and *B. oleracea* RFL proteins of the Brassica *Rf*-region. Numbers in red flanking each node indicate bootstrap values

We constructed a maximum-likelihood phylogeny to examine how the positional relationships among the various *RFL* genes reflected the sequence relatedness of the various encoded proteins. As shown in Fig. 7, all of the RFL proteins encoded in the Rf-syntenic regions of the different genomes formed a single monophyletic cluster encompassing the Arabidopsis subgroup 1 RFL proteins [22], and excluding radish Rfo/PPRA, their closest Arabidopsis homolog, AtRFL18, as well as the petunia Rf-PPR592 restorer protein and its non-restoring homolog (bootstrap support values of 0.90 and 1.00, respectively). Most of the Brassica proteins fell into a distinct cluster most closely related to the Arabidopsis branch containing *AtRFL1-3*. The exception were those proteins encoded by genes located at the same position as *AtRFL4* (At1g12700), PPR2 and Brara.I05097; these formed a distinct phylogenetic cluster (bootstrap support 1.00), suggesting that these Arabidopsis and Brassica proteins have descended from a common ancestor located at the same position in the ancestral genome.

Within the major Brassica clade, proteins in a common genomic location generally clustered together in the tree. The major exception concerned the *B. oleracea* genes Bol03187 and Bol03188. These proteins formed a cluster distinct from that of their positional counterparts, the *B. napus* PPR3 proteins, which clustered with PPR4 and its positional *B. oleracea* counterpart, Bol031408. An interesting situation is observed among the proteins encoded by Brara*.*I05115 and the genes at the corresponding positions in the *B. oleracea* and *B. napus* genomes, *B. oleracea* Bol03170 and Bol03171 and *Bn* 115. Although the Rf-region is derived from a *B. rapa* (A genome) ancestor, the *B. napus* and *B. rapa* 115 proteins each group with a different *B. oleracea* protein. Conceivably, orthologs of both genes were present in the common ancestor of the sequenced varieties of *B. napus* and *B. oleracea*, and different orthologs were lost during the subsequent evolution of the two A genome forms.

Our manual annotation of the portion of *A. lyrata* genome enriched in subgroup 1 *RFL* genes [22] led to the identification of 10 distinct genes. *AlyRFL1-10*, one of which (*AlyRFL10*) had too few PPR domains to merit inclusion in the phylogenetic tree. The two *A. lyrata* genes located at the position of *AtRFL2*/At1g12300 formed a distinct clade most closely related to the group of *A. thaliana RFL* genes encompassing *AtRFL2* as well as the closely related genes At*RFL3*/At1g12620 and At1g12775. The position of these two *lyrata* genes in the tree is consistent with a model in which they arose through a tandem duplication of an *AtRFL2-*like gene in an ancestral Arabidopsis genome, as proposed above. Similarly, *AlyRFL3*, *AlyRFL4* and *AlyRFL5* formed a monophyletic group with *AlyRFL6* and *AlyRFL8*, as would be predicted if there were a gene triplication followed by duplication of the three gene set. The exception to this model concerns *AlyRFL7*, which was predicted to cluster with *AlyRFL6* and *AlyRFL8* but instead groups with *AlyRFL9*. Interestingly, although the coding sequence of this *AlyRFL7* is more closely related to *AlyRFL9* than to *AlyRFL6/8*, more similarity is observed in the regions upstream and downstream of the gene to the corresponding sequences in the *AlyRFL3-5* region*.* Conceivably, *AlyRFL7* underwent a gene conversion event involving an *AlyRFL9* like sequence following duplication of the three gene region.

## Discussion

### *RFL* genes are dispersed in the Brassica Rf-region

A characteristic of genomic loci known to encode nuclear restorer proteins is the occurrence of multiple tandemly repeated related *RFL* genes [10–13, 15, 44]. Of these, only the rice locus encodes multiple Rf genes that suppress different forms of CMS. In contrast to other *Rf* loci, only two *RFL* genes within the *B. napus Rf* locus, *PPR1* and *PPR2*, are found adjacent to one another. Of these two, *PPR2* forms a phylogenetic group with *AtRFL4* that is distinct from that formed by the other Arabidopsis and Brassica *RFL* genes and thus represents a different line of evolutionary descent. Interestingly, the sequences flanking *AtRFL4* and Brassica *PPR2* are similar to one another, indicating that the genes have both descended from an ancestor located in the same genomic position.

### Retrotransposition has played a major role in the proliferation of Brassica Rf-region *RFL* genes

Comparison of the *B. napus Rf* locus to orthologous segments of related genomes has provided insight into the mechanisms by which this subgroup of *RFL* genes has expanded during the evolution of of Brassicacea genomes. With the exception of Brassica *PPR2*, these genes form a monophyletic cluster in the tree of Fig. 7, indicating descent from a common ancestor. None of the *B. napus/rapa RFL* genes share common 5’ or 3’ non-coding regions, suggesting that the expansion of this gene family occurred primarily through mechanisms involving retrotransposition. The observation that none of these genes contains an intron is consistent with this view.

Viewing the sequence relatedness among the different encoded proteins, as assessed through the phylogenetic tree, in the context of the positions of the genes on their respective genomes, provides additional insight into the mechanisms through which these genes proliferated. For example, *B. oleracea* Bol031408 and *B. napus/rapa PPR4*, cluster together on the tree, are located in corresponding positions on their genomes and have similar flanking sequences indicating these genes have descended from an ancestral gene present at that location in the last common ancestor of the A and C genomes. The coding sequences of *B. napus/rapa PPR3* form the closest neighboring phylogenetic cluster to these genes, but are present at a different genomic position, aligning with Bol03187/03188 and At1g12775. However, the sequences flanking *PPR3, PPR4,* Bol03188 and At1g12775 are all dissimilar, indicating that these genes have proliferated in their respective genomes through retrotransposition. Bol03187 and Bol03188 have similar flanking sequences, indicating a segmental duplication, but their coding sequences cluster with a Bol03184 and *PPR1*, suggesting that this duplication took place following retrotransposition of a *PPR1*-like gene.

### *RFL* gene proliferation in *A. thaliana* and *A. lyrata*

Apart from *AtRFL4*, discussed above, the Arabidopsis *RFL* genes found in genomic regions orthologous to the Brassica *Rf* locus form a monophyletic group, suggesting these genes have descended from ancestor(s) distinct from those that gave rise to the Brassica *Rf* locus *RFL* genes. Like majority the Brassica Rf-region *RFL* genes, the three *A. thaliana* proteins, AtRFL2, AtRFL3 and At1g12775, form a monophyletic cluster (bootstrap support values of 0.7–1.0), and are all located at different genomic positions and lack similarity in their flanking regions, indicating that their proliferation took place through retrotransposition events.

As discussed above, segmental duplication appears to have been the primary mechanism driving the duplication of this gene family in the *A. lyrata* lineage. Consistent with this model, three of the *A. lyrata* genes, *AlyRFL3-5*, predicted to be the products of tandem gene triplication, form a distinct cluster. Of the other three gene group predicted to arise from tandem duplication, *AlyRFL6-8*, *AlyRFL6* and *AlyRFL8* form a neighboring cluster in the tree, as would be expected if they arose from the duplication of the *AlyRFL3-5* region. *AlyRFL7*, however, clusters with *AlyRFL9*, which, with the very short *AlyRFL10* (not included in the phylogeny), was predicted to have been generated by a distinct duplication event. Interestingly, the sequences surrounding *AlyRFL7* are similar to those flanking *AlyRFL6* and *AlyRFL8,* as would be predicted from the segmental duplication model. It seems likely that following the duplication of the three gene region, retrotransposition and homologous recombination of an *AlyRFL9-*like gene led to the replacement of the coding sequence of the original gene located *AlyRFL7* site with a sequence resembling *AlyRFL9*.

### Prioritization of candidates for the *Rfn* gene

A key goal of this undertaking has been to identify candidates for the *Rfn* gene and to prioritize these candidates prior to proceeding with experiments aimed at rescuing the CMS and thereby conclusively identifying the gene. Since most restorer genes have been found to be *Rf-like PPR* genes and since the *Rfn* locus delimited in our mapping studies is enriched in RFL genes, we deem it most like that one of these genes functions as *Rfn.* Several other types of proteins have been suggested to play a role in fertility restoration, most notably Glycine-rich proteins, or GRPs [18, 44]. We did not, however, detect sequences capable of encoding such proteins in the genetically delimited Brassica *Rf* locus. Likely candidates for *Rfn* are therefore limited to the six *RFL* genes in this region.

It is logical that a restorer gene be expressed in floral tissues and because we were not able to obtain RT-PCR products for *Bn115* from floral RNA samples of either CMS or fertility restored plants, it seems unlikely to function as *Rfn. Bn036* is situated 100 kb from the flanking marker 4.4 BB but 300 kb from the next closest *RFL* gene and from the single marker that maps within the sequenced BAC and shows complete linkage to *Rfn*. It seems unlikely that the five recombination events we observed between 4.4BB and *Rfn* in the mapping experiments all occurred within the 100 kb interval between this marker and *Bn036. Bn036* is therefore judged not to be a strong candidate for *Rfn*. Of the genes within the selected BAC, we found through the analysis of transcripts of these genes that *PPR2* contains a premature termination codon in the line used as the *Rfn* parent of the mapping cross. Thus *PPR2* is unlikely to function as *Rfn*.

Thus, only *PPR1*, *PPR3* and *PPR4* are reasonable candidates for *Rfn*. Of these, *PPR4* is expressed in the restorer but not CMS parent an expression pattern previously observed in petunia restoring and non-restoring lines. While it remains possible that *PPR4* does not have a restorer function, it is interesting to note that this gene has also been proposed as a candidate for *Rfp* [42] and its absence of expression in the CMS line would explain why lines that can maintain the *nap* CMS can also serve as maintainers for *pol* CMS [29], i.e. they can serve as “universal” maintainers in *B. napus*. A unique and novel situation would arise if, indeed, different alleles of *PPR4* can function as restorers of different forms of CMS^2^.

## Conclusions

At the outset of this study, our goals were to map the *Rfn* region with sufficient resolution that we could further clarify the relationship of this gene with *Rfp*, the restorer for the other native CMS system in B*. napus*, and to identify a limited number of candidate genes that could then be tested for their capacity to rescue the *nap* CMS trait through transgenic complementation. It became evident that the Brassica *Rf* locus did not contain a group of tandemly repeated *RFL* genes, as do other characterized nuclear restorer loci, but that it did correspond to one of the *A. thaliana* and A*. lyrata* regions identified by Fujii and colleagues [22] that is enriched in a particular subgroup of *RFL* genes. We then sought a deeper understanding of the sequence relatedness of between *RFL* proteins encoded in the Brassica *Rf* locus and the Arabidopsis genomes. In particular we wanted to understand how the sequences of these proteins were related to their genomic position, and how this might relate to the mechanisms by which this group of genes evolved.

Our interpretation of the positional relationships among the genes, as illustrated in Fig. 4 and the phylogenetic relationship among the proteins, as illustrated in Fig. 7, is that both segmental duplication and retrotransposition processes each played a role in the evolution of this region, with segmental duplication being primarily responsible for the expansion of the family in *A. lyrata*, and retrotransposition playing a more major role in the Brassica genomes. Segmental duplication is most easily understood as arising through unequal genetic crossovers, but the mechanisms driving gene retrotransposition in plants are less well characterized. It has been suggested that the exchange of domains between PPR proteins may provide one means of functional diversification for RFL genes, but we were unable to observe any clear evidence for such an event. We did, however, find evidence for RNA-mediated gene conversion in which the coding sequence an *AlyRFL9*-like gene replaced the original central gene in the duplication involving the *AlyRFL6-8* genes.

Regardless of the mechanism through which this gene family has expanded in the different genomes, it is unclear what function many of these genes may have. AtRFL4 or RPF1, is known to specify specific RNA processing events, as do *Rfn* and *Rfp*. Post transcriptional processes and transcript stability control play a dominant role in determining the mature transcriptome of plant mitochondria. In this regard endonuclease cleavage provides a 3’ hydroxyl substrate for plant mitochondrial polyA polymerase, which targets substrates for degradation via polynucleotide phosphorylase [45]. The combined consequences of relaxed transcription and numerous ORFs of undefined function in plant mitochondria can result in the expression of “toxic” proteins. CMS provides the clearest example of this phenomenon. In this case, the function of the restorer protein can be viewed as specifying a site or sites for endonuclease cleavage that would then destabilize the 5’ end of the transcript and prevent its translation [29]. The cryptic transcripts that accumulate in mitochondrial polynucleotide phosphorylase mutants can include other ORFs [46], the expression of which may not cause male sterility but may be otherwise detrimental to the organism. The number and types of these ORFs can change rapidly and the transcript degradation system of the mitochondria must evolve rapidly to accommodate this change. It seems possible that this group of *RFL* proteins has evolved to specify novel endonuclease cleavage events that trigger the degradation of such transcripts.

## Methods

### Identification of an *Rfn* containing BAC

Primers based on the sequence of the cosmid 2840A3, generated from *Rfp* doubled haploid *B. rapa* containing the *Rfp*-linked SNP 12910 [35], were used for PCR-based screening [47] of a BAC library of *B. napus* doubled haploid line DH12075 constructed in the laboratory of Dr. Isobel Parkin, AAFC, Saskatoon, SK, Canada. A single BAC, designated NO202E11, was selected that generated amplification products with over 99 % similarity to cosmid 2840A3. This BAC contained the *Rfn*-associated allele of SNP 12910 and was therefore anchored in the *Rfn* genomic region. The purified BAC was nebulized, subjected to dideoxy sequencing and assembled at the McGill University – Génome Québec Innovation Centre.

### Annotation of the *Rfn* containing BAC sequence

The assembled sequence contained 8 non-overlapping contigs. These contigs were ordered using BLASTN (http://blast.ncbi.nlm.nih.gov/Blast.cgi) against *A. thaliana* and *B. rapa* genome sequences with the assumption that synteny of these genomes should be mainly conserved. Any missing sequences between the contigs and re-orientation of the sequences were investigated by using PCR to amplify the regions between the ends of each contig. Annotation of the completed sequence was investigated by first determining possible open reading frames using Genscan (http://genes.mit.edu/GENSCAN.html) (settings: organism: Arabidopsis, suboptimal exon cutoff: 1.0) and Softberry (http://www.softberry.com/berry.phtml?topic=case_study_plants&no_menu=on) (settings: organism: *Brassica rapa*) online-based tools. Subsequent analysis indicated that Softberry was more accurate at predicting ORFs and became the primary analytical tool for this purpose. Each predicted ORF was then used to find presumptive orthologs in the *Brassica rapa* and *Arabidopsis thaliana* genomes using the NCBI tool BLAST (http://blast.ncbi.nlm.nih.gov/Blast.cgi). This analysis allowed us to functionally categorize most of the genes within the BAC. Details of that analysis are presented in Additional file 2: Table S2.

### Plant growth and fertility scoring

Seeds from the two parental lines of *Brassica napus* used in this present study were acquired from Bo Gertsson, Lantmännen Lantbruk, Svalov, Sweden. The ‘Karat-Rfn’ nap restorer line is of Swedish origin that presents low levels of both erucic acid and glucosinolates. The population consisted of 318 BC_1_ plants derived from an intervarietal cross between single plants from nap CMS ‘Bronowski’ (*rfnrfn; nap*) and nap restorer ‘Karat-Rfn’ (*RfnRfn; nap*) lines. Seeds were plated onto basic Murashige and Skoog (Sigma-Aldridch M5524-10 L) medium and kept at 4 °C before germination in a chamber under standard light condition (16-h photoperiod, 22°/16 °C day/night temperatures) for a week. Seedlings were transferred in pots and grown to maturity in growth chambers or greenhouses under standard conditions (16-h photoperiod, 22°/16 °C day/night temperatures).

The fertility was assessed by the careful observation of five flowers per plant at least three times during the flowering period. The overall morphology of the flowers was noted as well as the production of pollen. The flower morphology at the earliest stages of development (2–3 days after flowering) was used as the main criteria to determine the fertility of plants segregating for the restoration of the *nap* CMS. Later in the development, some *nap* CMS flowers can produce pollen and make phenotyping ambiguous. Flowers from a male-fertility restored plant look identical to those of a fertile maintainer plant while flowers from a nap CMS plant have shrunken petals, and the style of the pistil is longer and often bent. CMS anthers of young flowers also have shorter filaments and no pollen or a reduced amount of pollen. The morphological contrast of young flowers between CMS and normal flowers was sufficient to allow plants carrying a restorer allele to be distinguished from those with only maintainer alleles, even though some CMS plants shed a small amount of pollen, especially in the later stages of development.

### Sampling, DNA extraction and SNP analysis

Two leaf disks from young plants were sampled into 96 well plates. DNA extraction and SNP genotyping was performed on a Sequenome platform by DNA LandMarks, Inc., St-Jean-sur-Richelieau, Quebec, Canada. 45 SNPs from a list of known *B. napus* polymorphisms, were selected on the basis of their position in of the on *B. rapa* chromosome A09 were screened for presence of polymorphism in *B. napus.* SNPs that were polymorphic between the parents of the cross were used to screen the entire BC1 population*.*

### Marker development

Analysis of the population was conducted in two phases. In the initial phase 293 plants were genotyped and phenotyped to roughly localize the *Rfn* gene. In the second stage, the *B. rapa* genome [48] was used to design ILP [38] and CAPS [39] markers to more precisely localize the gene. *B. rapa* gene sequences within the mapping interval predefined with the SNP analysis were retrieved from the phytozome database (http://phytozome.jgi.doe.gov/pz/portal.html). Primers were designed to amplify the first intron of every 5 genes using the primer3 web-based tool (bioinfo.ut.ee/primer3-0.4.0/). A 35 cycle PCR was performed following the supplier’s instruction (NEB M0273S). Size polymorphisms were detected on 2-3 % agarose gels. For the amplification products that did not show length polymorphisms, we attempted to identify CAPS by performing a 2 h digestion with enzyme HaeIII (recognition site GGCC) and AluI (recognition site AGCT) and running digestion products on a 2-3 % agarose gel. The amplification products for AFLPs and CAPS were cloned using the TOPO-TA cloning kit (Thermofisher K4575-01, Pleasanton, CA, USA). Information on the primers used, polymorphisms in the parent’s sequences can be found in Additional file 1: Table S1.

### Synteny analysis

*B. napus, B. rapa, B. oleracea* and *A. thaliana* genome fragments corresponding to the mapping interval were extracted from different online databases (https://genomevolution.org/coge/, http://phytozome.jgi.doe.gov/pz/portal.html, https://www.arabidopsis.org/index.jsp). The online tool PIP Maker (http://pipmaker.bx.psu.edu/pipmaker/) was used to assess the conservation of sequence linearity between the various genomes analyzed. *Brassica rapa* gene sequences were extracted (http://phytozome.jgi.doe.gov/pz/portal.html) in order to investigate homologs in *A. thaliana* (https://www.arabidopsis.org/index.jsp) and *B. napus* (https://genomevolution.org/coge/). Confirmation of co-linearity of relative position was obtained using the “Syntenic Gene” Search tool (http://brassicadb.org/brad/searchSyntenytPCK.php) of the BRAD online resource [43]. This analysis allowed the exploration of the synteny between the 3 genomes. Details of the data obtained are presented in Fig. 4.

### Identification of candidate genes and comparative genomics

Restorers of fertility inducing post-transcriptional processing, as *Rfn* does, have been characterized so far as part of the PPR P-type protein family. In order to do an exhaustive search of the *Rfn*-region *B. napus* genomic sequence for possible candidate genes, manual annotation of that genomic fragment was necessary. After extraction of the 600 Mb Rfn containing genomic sequence (http://brassicadb.org/cgi-bin/gbrowse/B.napus_chromosome/), ORF prediction using Softberry (http://www.softberry.com/berry.phtml?topic=case_study_plants&no_menu=on) with *B. rapa* prediction parameters was performed. For each ORF, a BLASTP search on the *Brassica rapa (*BRAD) and *Arabidopsis thaliana* (TAIR) genomes was performed in order to detect a probable homolog. That allowed gathering of information of the possible function of the predicted gene could be. With each predicted ORF presenting an annotation of PPR protein, PPR domain prediction was performed using TPRpred (http://toolkit.tuebingen.mpg.de/tprpred) with an e-value inclusion of 1e-6 as well as detection of a mitochondrial targeting site using TargetP (http://www.cbs.dtu.dk/services/TargetP/) (settings: plant organism group, no cutoff). Information on the syntenic regions from *B. rapa, B. oleracea and A. thaliana* were gathered from Gbrowse of the different genomic databases available for each genome (https://genomevolution.org/coge/, http://phytozome.jgi.doe.gov/pz/portal.html, https://www.arabidopsis.org/index.jsp). To identify corresponding genes in the *A. lyrata* genome, the region of the scaffold 1 assembly unit enriched in subgroup A_1 *RFL* [22] genes was manually annotated as described above.

### Expression of *Rfn* candidate genes

Expression of six candidate genes in CMS and restored flower buds were assessed RT-PCR. Total RNA extraction was first performed on 100 mg of floral tissue using Trizol (Thermofisher 15596–026, Pleasanton, CA, USA). Homogenization of frozen tissue was performed by grinding in a sterile mortar and pestle and adding 1 mL of Trizol. The mixture was mixed and then incubated 5 min at room temperature in order to allow rough extraction. 200 μL of chloroform was then added and the samples were mixed thoroughly for 15 s before incubation 2 min at room temperature and 4 °C centrifugation for 15 min at 13 000 g. The RNA extract in the upper aqueous phase was then precipitated with 0.5 mL isopropanol and incubated 10 min at room temperature before centrifuging at 4 °C at 12,000 g for 10 min. The supernatant was then rinsed with 1 mL 70 % ethanol and dissolved in 100 μL of RNAse free water at 55–60 °C for 10 min. Purification was then carried out with a standard Qiagen RNeasy kit (Cat No./ID 74904) according to the manufacturer’s instruction with DNAse treatment as suggested with (CatNo./ID 79254). After nanodrop quantification, reverse transcription was performed using SuperScript II reverse transcriptase according to the manufacturer instructions using 5 μg of total RNA. 2 μL of cDNA was used for a 35 cycles PCR using NEB Taq polymerase (catalog # M0273S, New England Biolabs, Ipswich, MA, USA) according to the supplier’s instructions with primers as described in Additional file 1: Table S1. No amplification of candidate *Bn115* was observed using samples from both the CMS and restored plants, suggesting this gene is not expressed, at least in the tissue analyzed. Multiple primer sets were tested with the same result. To analyze the presence of introns in the gene sequences the cDNA products were cloned using the TOPO-TA cloning kit (Thermofisher K4575-01) and sequencing was performed by ACGT (http://acgtcorp.com/, Toronto, ON, Canada) with the Applied BioSystems (ABI)/Life Technologies 3730xl capillary electrophoresis DNA sequencer. Sequence analysis of the delivered sequencing graphs were performed using the “geneious” program (http://www.geneious.com/).

### Phylogenetic analysis

From the different online databases available (https://genomevolution.org/coge/, http://phytozome.jgi.doe.gov/pz/portal.html, https://www.arabidopsis.org/index.jsp), we selected previously characterized restorer of fertility-like proteins from petunia and radish, and RFL proteins present in the regions syntenic to the *B. napus Rf* locus from *A. thaliana, B. rapa* and *B. oleracea*. For *A. lyrata*, sequences from scaffold 1 (between coordinates 4986000 and 5220000 based on the information provided in [22]) were extracted directly from JGI genome browser (http://genome.jgi.doe.gov/cgi-bin/browserLoad?db=Araly1&position=scaffold_1:1–100000) and screened for PPR proteins as described in the identification of *Rfn* candidates. The analysis of the *A. lyrata* sequence was also used for comparative genomics with *A. thaliana*. Protein sequences were appropriately formatted and phylogeny analysis were performed using LIRMM web based tool [49] (www.phylogeny.fr) in advanced mode, allowing a less stringent G Blocks selection and 400 boot strap in order to obtain a stable tree.

## Additional files

Additional file 1: Table S1. Information on molecular markers. (XLS 14 kb)
Additional file 2: Table S2. Annotation of B. napus BAC NO202E11. (XLS 20 kb)
Additional file 3: Table S3. Annotation of the B. napus 600 kb *Rf*-region. (XLS 52 kb)
Additional file 4: Figure S1. Phylogenetic analysis of proteins encoded in the *Rf*-region of *B. napus*/*rapa* and the orthologous segment of *A. thaliana.* Numbers in red type indicate bootstrap values. (PPTX 68 kb)
Additional file 5: Figure S2. Complementary DNA (cDNA) sequences of transcripts of *B. napus* genes *PPR1-4*. (PDF 36 kb)

## Abbreviations

CMS: Cytoplasmic male sterility
Mya: Million years ago or Million years before present
PPR: Pentatricopeptide repeat
*Rf*: Restorer-of-fertility
RFL: *Rf*-PPR-like Pentatricopeptide

## Footnotes

1 The *A. lyrata* genome region enriched in A_1 subgroup *RFL* genes was originally manually annotated by Fujii et al. [22] but it was not possible for us to determine the details of this annotation from the data provided. We therefore do not know the relationships between the *A. lyrata RFL* genes analyzed in [22] and those of derived from our annotation. For this reason, we have designated our genes and proteins as AlyRFL1, 2 etc. to distinguish them from those of the earlier publication which were designated as AlRFL1, 2 etc.

2 Subsequent to the submission of this manuscript, Liu and colleagues published evidence that the gene we refer to as PPR4, when isolated from the Rfp genotype, does indeed encode the Rfp gene (Molecular Plant 9:1082-1084, 2016).
