# Supplementary material for: Comparative genomic analysis of the compound Brassica napus Rf locus
Source: BMC Genomics. 2016 Oct 26;17:834. doi: 10.1186/s12864-016-3117-0 (PMC5080715; doi:10.1186/s12864-016-3117-0)
Supplement: Additional file 4: Figure S1. — Phylogenetic analysis of proteins encoded in the Rf-region of B. napus/rapa and the orthologous segment of A. thaliana. Numbers in red type indicate bootstrap values. (PPTX 68 kb) [file 12864_2016_3117_MOESM4_ESM.pptx]

## Slide 1
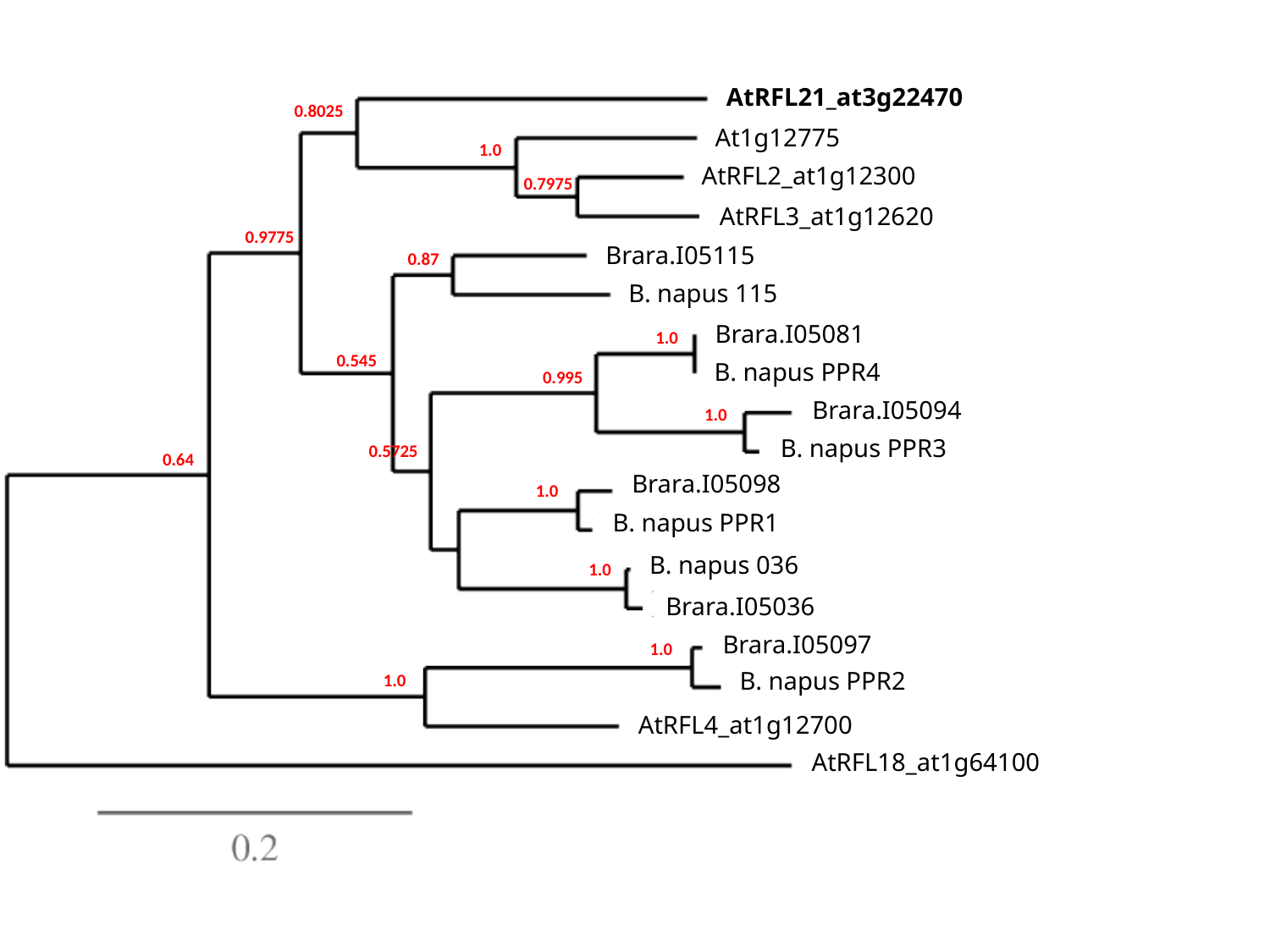

AtRFL21_at3g22470
At1g12775
AtRFL2_at1g12300
AtRFL3_at1g12620
Brara.I05115
B. napus 115
Brara.I05081
B. napus PPR4
Brara.I05094
B. napus PPR3
Brara.I05098
B. napus PPR1
B. napus 036
Brara.I05036
Brara.I05097
B. napus PPR2
AtRFL4_at1g12700
AtRFL18_at1g64100
0.8025
1.0
0.7975
0.9775
0.87
1.0
0.545
0.995
1.0
0.5725
0.64
1.0
1.0
1.0
1.0
