# Supplementary material for: Comparative genomic analysis of the compound Brassica napus Rf locus
Source: BMC Genomics. 2016 Oct 26;17:834. doi: 10.1186/s12864-016-3117-0 (PMC5080715; doi:10.1186/s12864-016-3117-0)
Supplement: Additional file 5: Figure S2. — Complementary DNA (cDNA) sequences of transcripts of B. napus genes PPR1-4. (PDF 36 kb) [file 12864_2016_3117_MOESM5_ESM.pdf]

PPR1 –

```
CMS     ATG ATG TTG ATG ATA AGG AGT GCC AAA GCT TTG AGA TCT GCT CGG CCT CTT CTC CTG GAG ACT GCA GGT ACC CTG AGA ACT TGT TTA CTC CAC AGC CCT TAC GAG TTC TCG TCT TTC
         M   M   L   M   I   R   S   A   K   A   L   R   S   A   R   P   L   L   L   E   T   A   G   T   L   R   T   C   L   L   H   S   P   Y   E   F  S/L  S   F
KARAT   ATG ATG TTG ATG ATA AGG AGT GCC AAA GCT TTG AGA TCT GCT CGG CCT CTT CTC CTG GAG ACT GCA GGT ACC CTG AGA ACT TGT TTA CTC CAC AGC CCT TAC GAG TTC TTG TCT TTC

CMS     GTT TGC GGA CGT GGC TTT TCT AGC AGC GAT AGA AAT
         V   C   G   R   G   F   S   S   S   D   R   N
KARAT   GTC TGC GGA CGT GGC TTT TCT AGC AGC GAT AGA AAT

CMS     CTC TGT TAT AGA GAG ACA TTG AGA AGT GGG CTC GTC GAT ATC AAG AAG GAT GAT GCT GTA GCT CTG TTT CAG TCC ATG GTT CGG TCT CGT CCT CTT CCT ACG GTC
         L   C   Y   R   E   T   L   R   S   G   L   V   D   I   K   K   D   D   A   V   A   L   F   Q   S   M  V/I  R   S   R   P   L   P   T   V
KARAT   CTC TGT TAT AGA GAG ACA TTG AGA AGT GGG CTC GTC GAT ATC AAG AAG GAT GAT GCT GTA GCT CTG TTT CAG TCC ATG ATT AGG TCT CGT CCT CTT CCT ACG GTC

CMS     ATA GAT TTC AAC AGA TTG TTT GGT TTA GTT GCC AAA ACG AAA CAG TAT GAC CTT GTC TTA GCT CTC TGC AAG CAA ATG GAA CTG AAA GGG ATT GCG TAT GAT CTA
         I   D   F   N   R   L   F   G   L   V   A   K   T   K   Q   Y   D   L   V   L   A   L   C   K   Q   M   E   L   K   G   I   A   Y   D   L
KARAT   ATA GAT TTC AAC AGA TTG TTT GGT TTA GTT GCC AAA ACG AAA CAG TAT GAC CTT GTC TTA GCT CTC TGC AAG CAA ATG GAA CTG AAA GGG ATT GCG TAT GAT CTA

CMS     TAC ACT CTC AAC ATT ATG ATC AAT TGC TTC TGC AGG CGT AGG AAA CTC GGT TTT GCT TTT TCT GCT ATG GGA AAG ATT TTG AAA CTT GGT TAT GAA CCT AGC ACG
         Y   T   L   N   I   M   I   N   C   F   C   R   R   R   K   L   G   F   A   F   S   A   M   G   K   I   L   K   L   G   Y   E   P   S   T
KARAT   TAC ACT CTC AAC ATT ATG ATC AAT TGC TTC TGC AGG CGT CGG AAA CTC GGT TTT GCT TTT TCT GCT ATG GGA AAG ATT TTG AAA CTT GGT TAT GAA CCT AGC ACG

CMS     ATC ACA TTC TCA ACT TTG ATT AAC GGA TTG TCT CTT GTG GGT AAA GTT TCT GAA GCT GTG GAG TTA GTT GAT CGA ATG GTG GGA ATG AAG GTT ATT CCA AAT CTC
         I   T   F   S   T   L   I   N   G   L  S/C  L  V/E  G  K/R V/L  S   E   A   V   E   L   V   D   R   M   V  G/E  M   K   V   I   P   N   L
KARAT   ATC ACA TTC TCA ACT TTG ATA AAC GGA TTG TGT CTT GAG GGT AGA CTC TCC GAA GCT GTG GAG TTA GTT GAT CGA ATG GTG GAA ATG AAG GTT ATT CCA AAT CTC

CMS     ATT ATA CTC AAC ACT ATT GTC AAT GGG CTT TGT CTC CAA GAC AGA TTG TCT GAA GCA ATG GCT TTG ATA GAT CGA ATG ATG GCC AAT GGA TGT CAA CCC GAC ACA
         I   I   L   N   T   I   V   N   G   L   C   L   Q   D   R   L   S   E   A   M   A   L   I   D   R   M   M   A   N   G   C   Q   P   D   T
KARAT   ATT ATA CTC AAC ACT ATT GTC AAT GGT CTT TGT CTC CAA GAC AGA TTG TCT GAA GCA ATG GCT TTG ATT GAT CGA ATG ATG GCT AAT GGA TGT CAA CCC GAC ACA

CMS     TTT ACC TAC GGT CCG GTT TTG AAC AGA ATG TGT AAG TCA GGG AAC ACT TCC TCC GCC TTG GAT CTG CTC AGA AAG ATG GAA GGT AGA AAA ATC GAA CTC GAT GCT
         F   T   Y   G   P   V   L   N   R   M   C   K   S   G   N   T   S   S   A   L   D   L   L   R  K/N  M   E   G   R   K   I   E   L   D   A
KARAT   TTT ACC TAC GGT CCG GTT TTG AAC AGA ATG TGT AAG TCA GGG AAC ACT TCC TCC GCC TTG GAT CTG CTC AGA AAC ATG GAA GGT AGA AAA ATC GAA CTC GAT GCT

CMS     GCT AAA TAC AAT GTC ATT ATT GAT AGT CTT TGC AAA GAT GGG AGC CTC GAC GAT GCA CTC ATC CTT TTC AAT GAA ATG GAA ACC AAA GGG GTC AAA GCA AAT GTC
         A   K   Y   N   V   I   I   D   S   L   C   K   D   G   S   L   D   D   A   L   I   L   F   N   E   M   E   T   K   G  V/I  K  A/P  N   V
KARAT   GCT AAA TAC AAT GTC ATT ATT GAT AGT CTT TGC AAA GAT GGG AGC CTC GAC GAT GCA CTC ATC CTT TTC AAT GAA ATG GAA ACC AAA GGG ATC AAA CCA AAT GTC

CMS     ATC ACC TAC AAC TCT CTC ATA GGA GGC TTC TGT AGT GCC GGC AGA TGG GAT GAT GGT GCA CAG CTG CTG AGG GAT ATG ATC ACA AGG GGA ATC ACC CCT AAC GTT
         I   T   Y   N   S   L   I   G   G   F   C   S   A   G   R   W   D   D   G   A   Q   L   L   R   D   M   I   T   R   G   I   T   P   N   V
KARAT   ATC ACC TAC AAC TCT CTC ATA GGA GGC TTC TGT AGT GCC GGC AGA TGG GAT GAT GGT GCA CAG CTG CTG AGG GAT ATG ATC ACA AGG GGA ATC ACC CCT AAC GTT

CMS     GTC ACT TTC AAT GCT TTG ATT GAT AGT TTT GTG AAA GAG GGA AAG CTT TCT GAG GCT GAA GAA TTG TAC AAT GAG ATG ACC CCA AGA GGA ATA GAT CCT AAT ACC
         V   T   F   N   A   L   I   D   S   F   V   K   E   G   K   L   S   E   A   E   E   L   Y   N   E   M  T/I  P   R   G   I   D   P   N   T
KARAT   GTC ACT TTC AAT GCT TTG ATT GAT AGT TTT GTG AAA GAG GGA AAG CTT TCT GAG GCT GAA GAA TTG TAC AAT GAG ATG ATC CCA AGA GGC ATA GAT CCT AAT ACT

CMS     ATT ACA TAT AGT ACT TTG ATA TAT GGG CTG TGC TAC GAA AAG CGC TTA GAT GAA GCC AAC CAG ATG CTG GAT CTG ATG GTT AGC AAG GGA TGC GAT CCT GAT ATT
         I   T   Y   S   T   L   I   Y   G   L   C   Y   E   K   R   L   D   E   A   N   Q   M   L   D   L   M   V   S   K   G   C   D   P   D   I
KARAT   ATT ACA TAT AGT ACT TTG ATA TAT GGG CTG TGC TAC GAA AAG CGC TTA GAT GAA GCC AAC CAG ATG CTG GAT CTG ATG GTT AGC AAG GGA TGC GAT CCT GAT ATT

CMS     TGG ACG TAT AAT ATC CTT ATA AAC GGG TAT TGT AAG GCT AAA CTG GTT GAT GAA GGT ATG AGA CTT TTC CGC AAA ATG TCT CTG AGA GGA GTG GTT GCA GAT ACA
         W   T   Y   N   I   L   I   N   G   Y   C   K   A   K   L   V   D  E/D  G   M   R   L   F   R   K   M   S   L   R   G   V   V   A   D   T
KARAT   TGG ACG TAT AAT ATC CTT ATA AAC GGG TAT TGT AAG GCT AAA CTG GTT GAT GAT GGT ATG AGA CTT TTC CGC AAA ATG TCT CTG AGA GGA GTG GTT GCA GAT ACA

CMS     GTC ACT TAT AGC AGT CTC ATT CAA GGG TTT TGT CAA TCA GGA AAA CTT AAA GTT GCC AAA GAA CTC TTC CAA GAG ATG GTT TCT GAA GGT GCT CAT CCT GAT ATT
         V   T   Y   S   S   L   I   Q   G   F   C   Q   S   G   K   L   K   V   A   K   E   L   F   Q   E   M   V   S   E   G   A   H   P   D   I
KARAT   GTC ACT TAT AGC AGT CTC ATT CAA GGG TTT TGT CAA TCA GGA AAA CTT AAA GTT GCC AAA GAA CTC TTC CAA GAA ATG GTT TCT GAA GGT GCT CAT CCT GAT ATT

CMS     GTG ACT TAC GGT ATT TTG CTG GAT GGT TTG TGT GAC AAT GGA GAA CTA GAG GAA GCC CTG GAA ATA CTT GAA AAA ATG CAC AAG ACT AAG ACG GAA CTT GAC ATT
         V   T   Y   G   I   L   L   D   G   L   C   D   N   G   E   L   E   E   A   L   E   I   L   E   K   M   H   K   T   K   T   E   L   D   I
KARAT   GTG ACT TAC GGT ATT TTG CTG GAT GGT TTG TGT GAC AAT GGA GAA CTA GAG GAA GCC CTG GAA ATA CTT GAA AAA ATG CAC AAG ACT AAG ACG GAA CTT GAC ATT

CMS     GGT ATA TAT AGT ATC ATC ATT CAC GGG ATG TGC AAT GCT AGT AAG GTC GAT GAT GCT TGG GAT CTG TTC TGC AGC CTA CCT TCG AAA GGA GTG AAG CCT GAT GTT
         G   I   Y   S   I   I   I   H   G   M   C   N   A   S   K   V   D   D   A   W   D   L   F   C   S   L   P   S   K   G   V   K   P   D   V
KARAT   GGT ATA TAT AGT ATC ATC ATT CAC GGG ATG TGC AAT GCT AGT AAG GTC GAT GAT GCT TGG GAT CTG TTC TGC AGC CTA CCT TCG AAA GGA GTG AAG CCT GAT GTT

CMS     AAG ACG TAC ACT GTA ATG ATT TCG GGA TTG TGT AAG AAA GGG TCA CTG CCT GAA GCA AAG ATG TTG CTT AGA AAA ATG GAG GAA GAT GGG ATT GCG CCA AAT GAT
         K   T   Y   T   V   M   I   S   G   L   C   K   K   G  S/L  L   P   E   A   K   M   L   L   R   K   M   E   E   D   G   I   A   P   N   D
KARAT   AAG ACG TAC ACT GTA ATG ATT TCG GGA CTG TGT AAG AAA GGG TTA CTG CCT GAA GCA AAG ATG TTG CTT AGA AAA ATG GAG GAA GAT GGG ATT GCG CCA AAT GAT

CMS     TGT ACA TAC AAC ACA CTA ATA CGA GCT CAT CTC AGA GGC AGC GAC ATA AGC AAT TCA GTT GAA CTC ATC GAA GAA ATG AAG AGG TGT GGC TTC TCT GCA GAT GCT
         C   T   Y   N   T   L   I   R   A   H   L   R   G   S   D   I   S   N   S   V   E   L   I   E   E   M   K   R   C   G   F   S   A   D   A
KARAT   TGT ACA TAC AAC ACA CTA ATA CGA GCT CAT CTC AGA GGC AGC GAC ATA AGC AAT TCA GTT GAA CTC ATC GAA GAA ATG AAG AGG TGT GGC TTC TCT GCA GAT GCT

CMS     TCC ACC ATG AAG ATG GTT ATG GAT ATG TTA TCG GAT GGT GGA TTG GAC AAA AGC TTT TTG GAT ATG CTT TCT TGA
         S   T   M   K   M   V   M   D   M   L   S   D   G   G   L   D   K   S   F   L   D   M   L   S   -
KARAT   TCC ACC ATG AAG ATG GTT ATG GAT ATG TTA TCG GAT GGT GGA TTG GAC AAA AGC TTT TTG GAT ATG CTT TCT TGA
```

PPR2 –

```
CMS      ATG TTG TTC TAC AGA AAG TCT ACC ACA CTT AAT CAA AAA GCT TCG AGA TTG GTT CAG CTT CAT CTC TCG GAG ACA GGT ACG CTT AGA ACT GAT TCG CTA TGT AGC TTC TCT ACC TTC
          M   L   F   Y  R/K K/M  S   T  T/A  L  N/H  Q   K   A   S   R   L   V   Q   L   H   L   S   E   T   G   T   L   R   T   D   S   L   C   S   F   S   T   F
KARAT    ATG TTG TTC TAC AAG ATG TCA ACC GCA CTT CAT CAA AAA GCT TCG AGA TTG GTT CAG CTT CAT CTC TCG GAG ACA GGT ACG CTT AGA ACT GAT TCG CTA TGT AGC TTC TCT ACC TTC
BAC      ATG TTG TTC TAC AAG ATG TCA ACC GCA CTT CAT CAA AAA GCT TCG AGA TTG GTT CAG CTT CAT CTC TCG GAG ACA GGT ACG CTT AGA ACT GAT TCG CTA TGT AGC TTC TCT ACC TTC

CMS      TTG TCT TGC TGC AAA CGA GAC TTC TCT GGA ATT ACC GAT GTG AAA
          L   S   C   C   K   R   D   F   S   G   I   T   D   V   K
KARAT    TTG TCT TGC TGC AAA CGA GAC TTC TCT GGA ATT ACC GAT GTG AAA
BAC      TTG TCT TGC TGC AAA CGA GAC TTC TCT GGA ATT ACC GAT GTG AAA

CMS      GTC TGT TTC AGA GAG AGA TTG AGG AAC GGA CTC GTC AAT ATC AAG AAA GAT GAT GCT GTT GCT CTC TTC CAA TCC ATG ATC AGG TCT AAT CCT CTT CCT ACA CTC
          V   C   F   R   E   R   L   R   N   G   L   V   N   I   K   K   D   D   A   V   A   L   F   Q   S   M   I   R   S   N   P   L   P   T   L
KARAT    GTC TGT TTC AGA GAG AGA TTG AGG AAC GGA CTC GTC AAT ATC AAG AAA GAT GAT GCT GTT GCT CTC TTC CAA TCC ATG ATC AGG TCT AAT CCT CTT CCT ACA CTC
BAC      GTC TGT TTC AGA GAG AGA TTG AGG AAC GGA CTC GTC AAT ATC AAG AAA GAT GAT GCT GTT GCT CTC TTC CAA TCC ATG ATC AGG TCT AAT CCT CTT CCT ACA CTC

CMS      ATC GAC TTC AGT AGA CTG TTC AGT GGT GTT GCC AAG ACA AAA CAG TAT GAT CTC GTG TTG AAT CTC TGC AAG CAA ATG GAA CTA AAC GGG ATT GCA CAT AAC ATC
          I   D   F   S   R   L   F   S   G   V   A   K   T   K   Q   Y   D   L   V   L   N   L   C   K   Q   M   E   L   N   G   I   A   H   N   I
KARAT    ATC GAC TTC AGT AGA CTG TTC AGT GGT GTT GCC AAG ACA AAA CAG TAT GAT CTC GTG TTG AAT CTC TGC AAG CAA ATG GAA CTA AAC GGG ATT GCA CAT AAC ATC
BAC      ATC GAC TTC AGT AGA CTG TTC AGT GGT GTT GCC AAG ACA AAA CAG TAT GAT CTC GTG TTG AAT CTC TGC AAG CAA ATG GAA CTA AAC GGG ATT GCA CAT AAC ATC

CMS      TAC ACT CTC AAC ATT ATG ATC AAC TGC TTT TGT CGT AGC TGC AGA ACT TGT TTT GCT TAC TCT GTT TTG GGG AAA GCT ATG AAG CTT GGG TTT AGC CCT GAC ACA
          Y   T   L   N   I   M   I   N   C   F   C   R   S   C   R   T   C   F   A   Y   S   V   L   G   K   A   M   K   L   G   F   S   P   D   T
KARAT    TAC ACT CTC AAC ATT ATG ATC AAC TGC TTT TGT CGT AGC TGC AGA ACT TGT TTT GCT TAC TCT GTT TTG GGG AAA GCT ATG AAG CTT GGG TTT AGC CCT GAC ACA
BAC      TAC ACT CTC AAC ATT ATG ATC AAC TGC TTT TGT CGT AGC TGC AGA ACT TGT TTT GCT TAC TCT GTT TTG GGG AAA GCT ATG AAG CTT GGG TTT AGC CCT GAC ACA

CMS      ACC ACA TAC AAC ACT CTC ATC AAT GGA CTC TGT CTT GAA GGC AAA GTC TCC GAA GCT GTG GGT TTG GTT AAT AAA ATG GTG GAG AAT GGA TGC CAA GCA GAC ACG
          T   T   Y   N   T   L   I   N   G   L   C   L   E   G   K   V   S   E   A   V   G   L   V   N   K   M   V   E   N   G   C   Q   A   D   T
KARAT    ACC ACA TAC AAC ACT CTC ATT AAT GGA CTC TGT CTT GAA GGC AAA GTC TCC GAA GCT GTG GGT TTG GTT AAT AAA ATG GTG GAG AAT GGA TGC CAA GCA GAC ACG
BAC      ACC ACA TAC AAC ACT CTC ATT AAT GGA CTC TGT CTT GAA GGC AAA GTC TCC GAA GCT GTG GGT TTG GTT AAT AAA ATG GTG GAG AAT GGA TGC CAA GCA GAC ACG

CMS      GTT ACG TTT GGT TCT ATA GTC AAT GGG ATT TGC AAA TCA GGA GAT ACT TCT CTG GCT TTG GAT TTT TTG AGG AAG ATG GAG GAA AGT GAT GTG AAG GCT GAT GTG
          V   T   F   G   S   I   V   N   G   I   C   K   S   G   D   T   S   L   A   L   D   F   L   R   K   M   E   E   S   D   V   K   A   D   V
KARAT    GTT ACG TTT GGT TCT ATA GTC AAT GGG ATA TGC AAA TCA GGA GAT ACT TCT CTG GCT TTG GAT TTT TTG AGG AAG ATG GAG GAA AGT GAT GTG AAG GCT GAT GTG
BAC      GTT ACG TTT GGT TCT ATA GTC AAT GGG ATA TGC AAA TCA GGA GAT ACT TCT CTG GCT TTG GAT TTT TTG AGG AAG ATG GAG GAA AGT GAT GTG AAG GCT GAT GTG

CMS      GTT ACG TAC AGT ACA GTT ATT GAT AGT CTT TGC AGA GAT GGG AGA ACG GAT GAT GCG GTT AAT CTA CTC AAT GAG ATG GAG AGG AAA GGA GTC AAG TCT AGT GTT
          V   T   Y   S   T   V   I   D   S   L   C   R   D   G   R   T   D   D   A   V   N   L   L   N   E   M   E   R   K   G   V   K   S   S   V
KARAT    GTT ACG TAC AGT ACA GTT ATT GAT AGT CTT TGC AGA GAT GGG AGA ACG GAT GAT GCG GTT AAT CTA CTC AAT GAG ATG GAG AGG AAA GGA GTC AAG TCT AGT GTT
BAC      GTT ACG TAC AGT ACA GTT ATT GAT AGT CTT TGC AGA GAT GGG AGA ACG GAT GAT GCG GTT AAT CTA CTC AAT GAG ATG GAG AGG AAA GGA GTC AAG TCT AGT GTT

CMS      GTT ACA TAT AAT TCT CTT GTA GGT GGG TTT TGT AAA GCT GGG AGA TGG
          V   T   Y   N   S   L   V   G   G   F   C   K   A   G   R   L
KARAT    GTT ACA TAT AAT TCT CTT GTA GGT GGG TTT TGT AAA GCT GGG AGA TGG
BAC      GTT ACA TAT AAT TCT CTT GTA GGT GGG TTT TGT AAA GCT GGG AGA TGG

CMS      GAT GAA GGT GCG AAG ATT TTG AAG GAT ATG ATT GGG AGG AAG ATG GTC CCT AAT GTT
          D   E   G   A   K   I   L   K   D   M   I   G   R   K   M   V   P   N   V
KARAT    GAT GAA GGT GCG AAG ATT TTG AAG GAT ATG ATT GGG AGG AAG ATG GTC CCT AAT GTT
BAC      GAT GAA GGT GCG AAG ATT TTG AAG GAT ATG ATT GGG AGG AAG ATG GTC CCT AAT GTT

CMS      GTG ACT TTC AAT GTG TTG ATT GAT GTT TGT GTT AAA GCA GGG AGG CTT GAN AAG GCT AAA GAG GTT TTA CGA GGA GAT GAT CAC GAG AGG TGT GGC TCC CAA TAC
          V   T   F   N   V   L   I   D   V   C   V   K   A   G   R   L   E   K   A   K   E   V   L   R   G   D   D   H   E   R   C   G   S   Q   Y
KARAT    GTG ACT TTC AAT GTG TTG ATT GAT GTT TGT GTC AAA GCA GGG AGG CTT GAG AAG GCT AAA GAG GTT TTA CGA GGA GAT GAT CAC GAG AGG TGT GGC TCC CAA TAC
BAC      GTG ACT TTC AAT GTG TTG ATT GAT GTT TGT GTC AAA GCA GGG AGG CTT GAG AAG GCT AAA GAG -TT TAC GAG GAG ATG ATC ACG AGA GGT GTG GCT CCC AAT ACT

CMS      TAT CAC TTA TAA CTC TTT GTG GTA GAT GGG TTT TGT ATG CAG AAC CGG CTC GGA GAG GCG AAG AAG ATG ATG GGT CTT ATG GTT GGG AGT AAC TGC AGT CCT GAT CTT
          Y   H   L   -   L  F/I  V   V   D   G   F   C   M   Q   N   R   L   G   E   A   K   K   M   M   G   L   M   V   G   S   N   C   S   P   D   L
KARAT    TAT CAC TTA TAA CTC ATT GTG GTA GAT GGG TTT TGT ATG CAG AAC CGG CTT GGA GAA GCG AAG AAG ATG ATG GGT CTT ATG GTT GGG AGT AAC TGC AGT CCT GAT CTT
BAC       ATC ACT TAT AAC TCA TT --G GTA GAT GGG TTT TGT ATG CAG AAC CGG CTT GGA GAA GCG AAG AAG ATG ATG GGT CTT ATG GTT GGG AGT AAC TGC AGT CCT GAT CTT

CMS      GTG ACT TTT AAT AGT CTC TTG AAA GGG TAT TGT AAG GTG AAA AGA GTT GAT GAT GCT ATG AAA CTC TTC AGG GAG TTT CCT GAG AGG GGA TTG GTT GCT AAT GAA
          V   T   F   N   S   L   L   K   G   Y   C   K   V   K   R   V   D  D/E  A   M   K   L   F   R   E   F   P   E   R   G   L   V   A   N   E
KARAT    GTG ACT TTT AAT AGT CTC TTG AAA GGG TAT TGT AAG GTG AAA AGA GTT GAT GAG GCT ATG AAA CTC TTC AGA GAG TTT CCT GAG AGG GGG TTG GTT GCT AAT GAA
BAC      GTG ACT TTT AAT AGT CTC TTG AAA GGG TAT TGT AAG GTG AAA AGA GTT GAT GAG GCT ATG AAA CTC TTC AGA GAG TTT CCT GAG AGG GGG TTG GTT GCT AAT GAA

CMS      GTT ACT TAT AGC ATC CTT GTT CAA GGG TTT TGT CAA TCC GGG AAA GTT AAG ATC GCT GAG GAG CTT TTT CAA GAA ATG GTT TCG TGT GGT GTT GTT CCT GAT GCT
          V   T   Y   S   I   L   V   Q   G   F   C   Q   S   G   K   V   K   I   A   E   E   L   F   Q   E   M   V   S   C   G   V   V   P   D   A
KARAT    GTT ACT TAT AGC ATT CTT GTT CAA GGG TTT TGC CAA TCC GGG AAA GTT AAG ATC GCT GAG GAG CTT TTT CAA GAA ATG GTT TCG TGT GGT GTT GTT CCT GAT GCT
BAC      GTT ACT TAT AGC ATT CTT GTT CAA GGG TTT TGC CAA TCC GGG AAA GTT AAG ATC GCT GAG GAG CTT TTT CAA GAA ATG GTT TCG TGT GGT GTT GTT CCT GAT GCT

CMS      ATG ACG TAT GGT ATA CTG CTT GAT GGT TTG TGT GAG AAC GGG AGG CTT GAG AAG GCG TTG GAG ATA TTT AAG GAT TTG GAA GAG AGT AAG ATG GAG CTT GAT GTT
          M   T   Y   G   I   L   L   D   G   L   C   E   N   G   R   L   E   K   A   L   E  I/M  F   K   D   L   E   E   S   K   M   E   L   D   V
KARAT    ATG ACG TAT GGT ATA TTG CTT GAT GGG TTG TGT GAG AAC GGG AGG CTT GAA AAG GCG TTG GAG ATG TTT AAG GAT TTG GAA GAG AGT AAG ATG GAG CTT GAT GTT
BAC      ATG ACG TAT GGT ATA TTG CTT GAT GGG TTG TGT GAG AAC GGG AGG CTT GAA AAG GCG TTG GAG ATG TTT AAG GAT TTG GAA GAG AGT AAG ATG GAG CTT GAT GTT

CMS      GTT ATG TAT ACG ATT ATG ATT GAG GAG ATG TGC AAG AGT GGT AAG GTG GAC GAT GCG TGG ACG TTG TTC TGT AGC CTA GGT TTG AAA GGA GTG AAG GCT AAT GTT
          V   M   Y   T   I   M   I   E  E/G  M   C   K   S   G   K   V   D   D   A   W   T   L   F   C   S   L   G   L   K   G   V   K   A   N   V
KARAT    GTT ATG TAT ACG ATT ATG ATT GAG GGG ATG TGC AAG AGT GGT AAG GTG GAT GAT GCT TGG ACG CTG TTC TGT AGC CTA GGT TTG AAA GGA GTG AAG GCT AAT GTT
BAC      GTT ATG TAT ACG ATT ATG ATT GAG GGG ATG TGC AAG AGT GGT AAG GTG GAT GAT GCT TGG ACG CTG TTC TGT AGC CTA GGT TTG AAA GGA GTG AAG GCT AAT GTT

CMS      AAG ACG TAC ACG GTG ATG ATT TGG GGA CTG TGT AAG AAA GGG TCG TTG TCT GAA GCA AAC ATG TTG CTT AGA AAA ATG GAG GAA GAT GGG AAT GCG CCG AAT GAT
         K/N  T   Y   T   V   M   I   W   G   L   C   K   K   G   S   L   S   E   A  N/K M/T  L   L   R   K   M   E   E   D   G   N   A   P   N   D
KARAT    AAT ACG TAC ACG GTG ATG ATT TGG GGA TTG TGT AAG AAA GGG TCG TTG TCT GAG GCA AAA ACG TTG CTT AGA AAA ATG GAG GAA GAT GGG AAT GCG CCG AAT GAT
BAC      AAT ACG TAC ACG GTG ATG ATT TGG GGA TTG TGT AAG AAA GGG TCG TTG TCT GAG GCA AAA ACG TTG CTT AGA AAA ATG GAG GAA GAT GGG AAT GCG CCG AAT GAT

CMS      TGT ACA TAC AAC ACT CTT GTC AGG GCA TAT CTT CGA GAT TGC GAC TTA GCC AAA TCA GCA GAA CTT ATA GAA GAA ATG AAG AGT TAT GGG TTC TCA GCA GAT GCG
          C   T   Y   N   T   L   V   R   A   Y   L   R  D/E  C   D   L   A   K   S   A   E   L   I   E   E   M   K   S   Y   G   F   S   A   D   A
KARAT    TGT ACA TAC AAC ACT CTT GTC AGG GCA TAT CTT CGA GAG TGC GAC TTA GCC AAA TCA GCA GAA CTT ATT GAA GAA ATG AAG AGT TAT GGG TTC TCA GCA GAT GCG
BAC      TGT ACA TAC AAC ACT CTT GTC AGG GCA TAT CTT CGA GAG TGC GAC TTA GCC AAA TCA GCA GAA CTT ATT GAA GAA ATG AAG AGT TAT GGG TTC TCA GCA GAT GCG

CMS      TCC ACT GTT AAG ATG GTG ATG GAT AGG TTA TCT AGC GGT GAA TTG GAT AAA AGA TTT TTG
          S   T   V   K   M   V   M   D   R   L   S   S   G   E   L   D   K   R   F   L
KARAT    TCC ACT GTT AAG ATG GTG ATG GAT AGG TTA TCT AGC GGT GAA TTG GAT AAA AGA TTT TTG
BAC      TCC ACT GTT AAG ATG GTG ATG GAT AGG TTA TCT AGC GGT GAA TTG GAT AAA AGA TTT TTG

CMS      GAT ATG CTC TCT TAG
          D   M   L   S   -
KARAT    GAT ATG CTC TCT TAG
BAC      GAT ATG CTC TCT TAG
```

PPR3 -

```
CMS      GAG ACG ATG ATG TTG ATG ATT CGG AGT GCC AAA GCT TTG AGA TCT GTT CGG CCT
         E/M T/M M/L  M  L/I M/R  I   R   S   A   K   A   L   R   S   V   R   P
KARAT    ATG ATG TTG ATG ATT CGG ATA AGG AGT GCC AAA GCT TTG AGA TCT GTT CGG CCT

CMS      CGA TTC ATG GAG ACA GCA GGT ACC CTG AGA ATT TCT CTA TTC CAC AGA ACC CCA TAC GAG
          R   F   M   E   T   A   G   T   L   R   I   S   L   F   H   R   T   P   Y   E
KARAT    CCA TTC ATG GAG ACA GCA GGT ACC CTG AGA ATT TCT CTA TTC CAC AGA ACC CCA TAC GAG

CMS      CTC TTG TCT TTC GTC TGC GAA AGA ACC TTC TCT GGT GGT AGC GAT AGA AAG ATG TCT GCT
          L   L   S   F   V   C   E   R   T   F   S   G   G   S   D   R   K   M   S   A
KARAT    CTC TTG TCT TTC GTC TGC GAA AGA ACC TTC TCT GGT GGT AGC GAT AGA AAG ATG TCT GCT

CMS      GCT TCT TAC AAA GAG AGA CTG AGA AGT GGG ATT ATT GGT ATC AAG AAG GAT GAA GCT GTT GCT CTG TTT CAG TCC ATG ATT AGG TCT CGC CCT CTT CCA ACA ATC
          A   S   Y   K   E   R   L   R   S   G   I   I   G   I   K   K   D   E   A   V   A   L   F   Q   S   M   I   R   S   R   P   L   P   T   I
KARAT    GCT TCT TAC AAA GAG AGA CTG AGA AGT GGG ATT ATT GGT ATC AAG AAG GAT GAA GCT GTT GCT CTG TTT CAG TCC ATG ATT AGG TCT CGC CCT CTT CCA ACA ATC

CMS      ATA GAT TTC AAC AGA TTG TTT ACT GCA ATG GCC AAA ACA AAA CAG TAT GAT CTC GTG TTG GAT CTC TGC AAG CAG ATG GAG TTG AAT GGG ATT GCA CAT AAC ATC
          I   D   F   N   R   L   F   T   A   M   A   K   T   K   Q   Y   D   L   V   L   D   L   C   K   Q   M   E   L   N   G   I   A   H   N  I/M
KARAT    ATA GAT TTC AAC AGA TTG TTT ACT GCA ATG GCC AAA ACA AAA CAG TAT GAT CTC GTG TTG GAT CTC TGC AAG CAG ATG GAG TTG AAC GGG GTT GCA CAT AAC ATG

CMS      TAC ACT CTC AAC ATT ATG ATC AAT TGC TTC TGC AGG CGT CCT AAA CTT GGT TTT GCT TTT TCT GTG ATG GGA AAG ATG TTG AAG CTT GGT TAT GAG CCC GAC AGA
          Y   T   L   N   I   M   I   N   C   F   C   R   R  P/W  K   L   G   F   A   F   S   V   M   G   K  M/I  L   K   L   G   Y   E   P   D   R
KARAT    TAC ACT CTC AAC ATT ATG ATC AAT TGC TTC TGC CGT CGC TGG AAA CTC GGT TTT GCT TTC TCT GTG ATG GGA AAG ATC TTG AAG CTT GGT TAT GAG CCC GAC AGA

CMS      GTC ACA TTT AAC ACC CTT CTC AAT GGC TTA TGT CTC GAG GGT AGA GTC TTT GAC GAT GTG GAG TTA GTT GAT TGT ATG GTC CTA AGC CAA CAT GTA CCA GAT CTC
          V   T   F   N   T   L   L   N   G   L   C   L   E   G   R   V   F   D  D/A  V   E   L   V   D   C   M   V   L   S   Q   H   V   P   D   L
KARAT    GTC ACA TTT AAC ACC CTT CTC AAT GGT TTA TGT CTC GAG GGT AGA GTC TTT GAC GCT GTG GAG TTA GTT GAT TGT ATG GTC CTA AGC CAA CAT GTA CCA GAT CTC

CMS      ATC ACC CTC AAC ACT CTT GTC AAT GGA CTT TGT CTC AAA GAT AGA GTC TCT GAA GCA GTG GAT TTA ATA GCT CGA ATG ATG GAT AAA GGA TGT CTA GCC GAT CAG
          I   T   L   N   T   L   V   N   G   L   C   L   K   D   R   V   S   E   A   V   D   L   I   A   R   M   M   D   K   G   C   L   A   D   Q
KARAT    ATC ACC CTC AAC ACT CTT GTC AAT GGA CTT TGT CTC AAA GAT AGA GTC TCT GAA GCA GTG GAT TTA ATA GCT CGA ATG ATG GAT AAA GGA TGT CAA GCC GAT CAG

CMS      TTT ACC TAT GGT CCG ATC TTG AAC AGA ATG TGT AAG TCA GGG AAC ACT ACA TTG GCC TTG GAT CTA CTC ACA AAG ATG GAA GAT AGA AAA GTC AAG CCT CAC GTA
          F   T   Y   G   P   I   L   N   R   M   C   K  S/F  G   N   T   T   L   A   L   D   L   L   T   K   M   E   D   R   K   V   K   P   H   V
KARAT    TTT ACC TAT GGT CCA ATC TTG AAC AGA ATG TGT AAG TTT GGG AAC ACT ACA TTG GCC TTG GAT CTA CTC ACA AAG ATG GAA GAT AGA AAA GTC AAG CCT CAC GTA

CMS      GTC ACA TAC AAT ATT ATT ATC GAT AGT CTT TGC AAA GAT GGG AGC CTC GAC GAT GCA CTC AGC TTT TTC AGT GAA ATG GAA ACC AAA GGG ATC AAA GCA GAT GTC
          V   T   Y   N   I  I/V  I   D   S   L   C   K   D   G   S   L   D   D   A   L   S   F   F   S   E   M   E   T   K   G   I   K   A   D   V
KARAT    GTC ACA TAC AAT ATT GTT ATC GAT AGT CTT TGC AAA GAT GGG AGC CTC GAC GAT GCA CTC AGC TTT TTC AGT GAA ATG GAA ACC AAA GGG ATC AAA GCA GAT GTC

CMS      TTT ACC TAC ACC TCT CTC ATA GGA GGC TTC TGT AGT GTT GGT AAA TGG GAT GAT GGT GCA CAG TTG CTG AGG GAT ATG ATT CGA AGG GGA ATC ACC CCG AAC GCT
          F   T   Y   T   S   L   I   G   G   F   C   S   V   G   K   W   D   D   G   A   Q   L   L   R   D   M   I   R   R  G/E  I   T   P   N   A
KARAT    TTT ACC TAC ACC TCT CTC ATA GGA GGC TTC TGT AGT GTT GGT AAA TGG GAT GAT GGT GCA CAG TTG CTG AGG GAT ATG ATT CGA AGG GAA ATC ACC CCG AAC GCC

CMS      ATC ACT TTC AGT TCT TTG ATT GAT AGT TTT GTG AAA GTG GGA AAG CTT TCT GAG GCT CAA GAT CTG TAC AAC GAG ATG ATC AAA AGA GGC ACA GAT CCT GAC ACC
          I   T   F   S   S   L   I   D   S   F   V   K   V   G   K   L   S   E   A   Q   D   L   Y   N   E   M   I   K   R   G   T   D   P   D   T
KARAT    ATC ACT TTC AGT TCT TTG ATT GAT AGT TTT GTG AAA GTG GGA AAG CTT TCT GAG GCT CAA GAT CTG TAC AAC GAG ATG ATC AAA AGA GGC ACA GAT CCT GAC ACC

CMS      ATT ACA TAT AAT TCT TTG ATA TAT GGG TTG TGC ATG GAG AAG CGC TTA GAT GAG GCC AGA GAG ATG CTG GAT CTG ATG GTT AGC AAG GGA TGT GAT CCA GAT ATT
          I   T   Y   N   S   L   I   Y   G   L   C   M   E   K   R   L   D   E   A   R   E   M   L   D   L   M   V   S   K   G   C   D   P   D   I
KARAT    ATT ACA TAT AAT TCT TTG ATA TAT GGG TTG TGC ATG GAG AAG CGC TTA GAT GAG GCC AGA GAG ATG CTG GAT CTG ATG GTT AGC AAG GGA TGT GAT CCA GAT ATT

CMS      GTG ACT TAT AGT ATC CTT ATA AAC GGC TAC TGC AAG GCT AAA CTG GTT GAT GAA GGT ATG AGA CTT TTC CGC AAA ATG ACC TTG AGA GGG GTG GTT GCC AAT ACA
          V   T   Y   S   I   L   I   N   G   Y   C   K   A   K   L   V   D   E   G   M   R   L   F   R   K   M   T   L   R   G   V   V   A   N   T
KARAT    GTG ACT TAT AGT ATC CTT ATA AAC GGC TAC TGC AAG GCT AAA CTG GTT GAT GAA GGT ATG AGA CTT TTC CGC AAA ATG ACC TTG AGA GGG GTG GTT GCC AAT ACA

CMS      GTG ACT TAT AGC ACT CTC ATC CAA GGG TTT TGT CAA TCT GGA AAA CTT AAT GTT GCC AAA GAA CTC TTC CAG GAG ATG GTT TCT GAA GGT GTT CGT CCT AGT ATT
          V   T   Y   S   T   L   I   Q   G   F   C   Q   S   G   K   L   N   V   A   K   E   L   F   Q   E   M   V   S   E   G   V  R/H  P   S   I
KARAT    GTG ACT TAT AGC ACT CTC ATC CAA GGG TTT TGT CAA TCT GGA AAA CTT AAT GTT GCC AAA GAA CTC TTC CAG GAG ATG GTT TCT GAA GGT GTT CAT CCT AGT ATT

CMS      ATG ACT TAC GGT ATT TTG CTG GAT GGG TTG TGT GAC AAT GGA GAA GTA GAG GAA GCC ATG GAA ATA CTT GAA AAA ATG CAC AAG TGT AAG ATT GAT CCT GGT ATT
          M   T   Y   G   I   L   L   D   G   L   C   D   N   G   E  V/L  E   E   A  M/L E/G  I   L   E   K   M   H   K   C   K   I   D   P   G   I
KARAT    ATG ACT TAC GGT ATT TTG CTG GAT GGG TTG TGT GAC AAT GGC GAA CTA GAA GAG GCT TTG GGA ATA CTT GAA AAA ATG CAC AAG TGT AAG ATT GAT CCT GGT ATT

CMS      GGT ATA TAT ACT ATC ATC ATT CAC GGT ATG TGC AAT GCA AAT AAG GTC GAT GAT GCT TGG GAT CTA TTC TGC AGC CTC TCT CTC AAA GGA GTG AAG CGT GAT ATT
          G   I   Y   T   I   I   I   H   G   M   C   N   A   N   K  V/I  D   D   A   W   D   L   F   C   S   L   S   L   K   G   V   K   R   D   I
KARAT    GGT ATC TAT ACT ATC ATC ATT CAC GGT ATG TGC AAT GCA AAT AAG ATC GAT GAT GCT TGG GAT CTA TTC TGC AGC CTC TCT CTC AAA GGA GTG AAG CGT GAT ATT

CMS      CGG TCA TAC AAC ATA ATG TTG TCA GGA TTA TGT AAG AGG AGC TCA TTG TCT GAA GCG GAT GCA TTG TTT AGA AAA ATG AAG GAA GAT GGG TAT GAG CCA GAT GAT
          R   S   Y   N   I   M   L   S   G   L   C   K   R   S   S   L   S   E   A   D   A   L   F   R   K   M   K   E   D   G   Y   E   P   D   D
KARAT    CGG TCA TAC AAC ATA ATG TTG TCA GGA TTA TGT AAG AGG AGC TCA TTG TCT GAA GCG GAT GCA TTG TTT AGA AAA ATG AAG GAA GAT GGG TAT GAG CCA GAT GAT

CMS      TGT ACG TAC AAT ACA CTT ATC AGA GCA CAT CTT CGA GGT AGT GAC ATA ACA ACT TCA GTT CAA CTC ATT GAA GAA ATG AAG AGG TGT GGG TTC TCT TCA GAT GCT
          C   T   Y   N   T   L   I   R   A   H   L   R   G   S   D   I   T   T   S   V   Q   L   I   E   E   M   K   R   C   G   F   S   S   D   A
KARAT    TGT ACG TAC AAT ACA CTT ATC AGA GCA CAT CTG CGA GGT AGT GAC ATA ACA ACT TCA GTT CAA CTC ATT GAA GAA ATG AAG AGG TGT GGG TTC TCT TCA GAT GCT

CMS      TCC ACC GTA AAG ATT GTT ATG GAT ATG CTA TCG AGT GGT GAA TTG GAC AAA AGC TTT CTA GAT
          S   T   V   K   I   V   M   D   M   L   S   S   G   E   L  D/N  K   S   F   L   D
KARAT    TCC ACC GTA AAG ATT GTT ATG GAT ATG CTA TCG AGT GGT GAA TTG AAC AAA AGC TTT CTA GAT

CMS      ATG CTT TCT GGT CCT TCT CGA GAG ATA GCA TCA TCG TTG GAT TGA
          M   L   S   G   P   S   R   E   I   A   S   S   L   D   -
KARAT    ATG CTT TCT GGT CCT TCT CGA GAG ATA GCA TCA TCG TTG GAT TGA
```

PPR4 -

```
CMS     ATG GTG TTG AGG ACA CAG AGA TGG
         M   V   L   R   T   Q   R   W
KARAT   ATG GTG TTG AGG ACA CAG AGA TGG

CMS     AAT CGT CTT ACT ACT TTG AGA TTG GTT CAT CTC CGT TCA ACT GAG ACA GGT ACT CTG AGA
         N   R   L   T   T   L   R   L   V   H   L   R   S   T   E   T   G   T   L   R
KARAT   AAT CGT CTT ACT ACT TTG AGA TTG GTT CAT CTC CGT TCA ACT GAG ACA GGT ACT CTG AGA

CMS     AAT GCT GCT TTC TTC CAA AGC CCA TAC GAC TTC TTC TTC TGC GTA CAA GGC TTC TCT GGT
         N   A   A   F   F   Q   S   P   Y   D   F   F   F   C   V   Q   G   F   S   G
KARAT   AAT GCT GCT TTC TTC CAA AGC CCA TAC GAC TTC TTC TTC TGC GTA CAA GGC TTC TCT GGT

CMS     CTC ACC AGC GAT AGA AAG ATG
         L   T   S   D   R   K   M
KARAT   CTC ACC AGC GAT AGA AAG ATG

CMS     TCT TCT TAC AAA GAG AGA TTG AGA AGT GGT CTC GTC GAT ATC AAG AAG GAT GAT GCT GTT GCT CTG TTT CAG TCC ATG CTT CGG TCT CGT CCT CTT CCT ACG GTC
         S   S   Y   K   E   R   L   R   S   G   L   V   D   I   K   K   D   D   A   V   A   L   F   Q   S   M   L   R   S   R   P   L   P   T   V
KARAT   TCT TCT TAC AAA GAG AGA TTG AGA AGT GGT CTC GTC GAT ATC AAG AAG GAT GAT GCT GTT GCT CTG TTT CAG TCC ATG CTT CGG TCT CGT CCT CTT CCT ACG GTC

CMS     ATT GAT TTC AAC AGA TTG TTT GGT TTA CTT GCC AGA ACT AAA CAG TAC GAT CTC GTG TTA GCT CTC TGC AAG CAA ATG GAA CTG AAA GGG ATT GCG TAT GAC CTC
         I   D   F   N   R   L   F   G   L   L   A   R   T   K   Q   Y   D   L   V   L   A   L   C   K   Q   M   E   L   K   G   I   A   Y   D   L
KARAT   ATT GAT TTC AAC AGA TTG TTT GGT TTA CTT GCC AGA ACT AAA CAG TAC GAT CTC GTG TTA GCT CTC TGC AAG CAA ATG GAA CTG AAA GGG ATT GCG TAT GAC CTC

CMS     TAC ACT CTC AAC ATT ATG ATC AAT TGC TTC TGC AGG CGT CGG AAA CTC GGT TTT GCT TTT TCC GCT ATG GGG GAG ATC TTC AAA CTT GGG TAT GAG CCT AAC ACA
         Y   T   L   N   I   M   I   N   C   F   C   R   R   R   K   L   G   F   A   F   S   A   M   G   E   I   F   K   L   G   Y   E   P   N   T
KARAT   TAC ACT CTC AAC ATT ATG ATC AAT TGC TTC TGC AGG CGT CGG AAA CTC GGT TTT GCT TTT TCC GCT ATG GGG GAG ATC TTC AAA CTT GGG TAT GAG CCT AAC ACA

CMS     GTC ACA TTT AAC ACC CTC CTC AAT GGC TTA TGT CTC GAG GGC AGA GTC TTT GAA GCT GTG GAG TTA GTT GAT TGT ATG GTC CTA AGC CAA CAT GTA CCA GAT CTT
         V   T   F   N   T   L   L   N   G   L   C   L   E   G   R   V   F   E   A   V   E   L   V   D   C   M   V   L   S   Q   H   V   P   D   L
KARAT   GTC ACA TTT AAC ACC CTC CTC AAT GGC TTA TGT CTC GAG GGC AGA GTC TTT GAA GCT GTG GAG TTA GTT GAT TGT ATG GTC CTA AGC CAA CAT GTA CCA GAT CTT

CMS     ATC ACC CTC AAC ACT ATT GTC AAT GGG CTT TGT CTC AAA GAT AGA GTG TCT GAA GCA GTG GAT TTA ATA GCT CGA ATG ATG GAT AAA GGA TGT CAA GCC GAT CAG
         I   T   L   N   T   I   V   N   G   L   C   L   K   D   R   V   S   E   A   V   D   L   I   A   R   M   M   D   K   G   C   Q   A   D   Q
KARAT   ATC ACC CTC AAC ACT ATT GTC AAT GGG CTT TGT CTC AAA GAT AGA GTG TCT GAA GCA GTG GAT TTA ATA GCT CGA ATG ATG GAT AAA GGA TGT CAA GCC GAT CAG

CMS     TTT ACC TAT GGT CCA ATC TTG AAC AGA ATG TGT AAG TCT GGG AAC ACT GCC TCG GCC TTG GAT CTG CTC AGG AAG ATG GAA CAT AGA AAG ATC AAG CCA CAC GTA
         F   T   Y   G   P   I   L   N   R   M   C   K   S   G   N   T   A   S   A   L   D   L   L   R   K   M   E   H   R   K   I   K   P   H   V
KARAT   TTT ACC TAT GGT CCA ATC TTG AAC AGA ATG TGT AAG TCT GGG AAC ACT GCC TCG GCC TTG GAT CTG CTC AGG AAG ATG GAA CAT AGA AAG ATC AAG CCA CAC GTA

CMS     GTC ACA TAC AAT ATC ATC ATT GAC AAT CTT TGC AAA GAT GGG AGA CTC GAC GAT GCA CTC AGC TTT TTC AGT GAA ATG GAA ACC AAA GGG ATC AAA GCA GAT GTC
         V   T   Y   N   I   I   I   D   N   L   C   K   D   G   R   L   D   D   A   L   S   F   F   S   E   M   E   T   K   G   I   K   A   D   V
KARAT   GTC ACA TAC AAT ATC ATC ATT GAC AAT CTT TGC AAA GAT GGG AGA CTC GAC GAT GCA CTC AGC TTT TTC AGT GAA ATG GAA ACC AAA GGG ATC AAA GCA GAT GTC

CMS     ATT ACC TAC AAC TCT CTC ATA GGA AGC TTC TGT AGT TTT GGC AGA TGG GAT GAT GGT GCA CAG TTG CTG AGG GAT ATG ATT ACA AGG AAA ATC ACC CCC AAC GTT
         I   T   Y   N   S   L   I   G   S   F   C   S   F   G   R   W   D   D   G   A   Q   L   L   R   D   M   I   T   R   K   I   T   P   N   V
KARAT   ATT ACC TAC AAC TCT CTC ATA GGA AGC TTC TGT AGT TTT GGC AGA TGG GAT GAT GGT GCA CAG TTG CTG AGG GAT ATG ATT ACA AGG AAA ATC ACC CCC AAC GTT

CMS     GTC ACT TTC AGT GCT TTG ATT GAT AGT CTT GTT AAA GAG GGA AAG CTT ACT GAG GCT AAA GAC TTG TAC AAT GAG ATG ATC ACA AGA GGC ATA GAT CCT AAT ACC
         V   T   F   S   A   L   I   D   S   L   V   K   E   G   K   L   T   E   A   K   D   L   Y   N   E   M   I   T   R   G   I   D   P   N   T
KARAT   GTC ACT TTC AGT GCT TTG ATT GAT AGT CTT GTT AAA GAG GGA AAG CTT ACT GAG GCT AAA GAC TTG TAC AAT GAG ATG ATC ACA AGA GGC ATA GAT CCT AAT ACC

CMS     ATT ACA TAT AGT ACT TTG ATA TAT GGG TTG TGC ATG GAG AAC CGC TTA GAT GAA GCC AAC CAG ATG ATG GAC CTC ATG GTT AGC AAG GGA TGC GAT CCT GAT ATC
         I   T   Y   S   T   L   I   Y   G   L   C   M   E   N   R   L   D   E   A   N   Q   M   M   D   L   M   V   S   K   G   C   D   P   D   I
KARAT   ATT ACA TAT AGT ACT TTG ATA TAT GGG TTG TGC ATG GAG AAC CGC TTA GAT GAA GCC AAC CAG ATG ATG GAC CTC ATG GTT AGC AAG GGA TGC GAT CCT GAT ATC

CMS     GTG ACG TTT AAT GTC CTT ATA AAC GGA TTT TGT AAG GCT AAA CAG GTT GAT GTT GGT ATG AGA CTA TTC CGA AAG ATG TCT CTG AGA GGA GAG ATT GCA GAT ACA
         V   T   F   N   V   L   I   N   G   F   C   K   A   K   Q   V   D   V   G   M   R   L   F   R   K   M   S   L   R   G  E/V  I   A   D   T
KARAT   GTG ACG TTT AAT GTC CTT ATA AAC GGA TTT TGT AAG GCT AAA CAG GTT GAT GTT GGT ATG AGA CTA TTC CGA AAG ATG TCT CTG AGA GGA GTG ATT GCA GAT ACA

CMS     GTG ACT TAT AGC ACT CTC ATC CAA GGG TTT TGT CAA TCA AGA GAA CTT ATT GTC GCC AAA GAG GTC TTC CAA GAG ATG GTC TCT CAA GGT GTT CAT CCT GGT ATT
         V   T   Y   S   T   L   I   Q   G   F   C   Q   S   R   E   L   I   V   A   K   E   V   F   Q   E   M   V   S   Q   G   V   H   P   G   I
KARAT   GTG ACT TAT AGC ACT CTC ATC CAA GGG TTT TGT CAA TCA AGA GAA CTT ATT GTC GCC AAA GAG GTC TTC CAA GAG ATG GTC TCT CAA GGT GTT CAT CCT GGT ATT

CMS     ATG ACT TAT GGT ATT TTG CTG GAT GGG TTG TGT GAC AAT GGC GAA CTA GAA GAG GCT TTG GGA ATA CTT GAT CAA ATG CAC AAG TGT AAG ATG GAA CTT GAT ATT
         M   T   Y   G   I   L   L   D   G   L   C   D   N   G   E   L   E   E   A   L   G   I   L   D   Q   M   H   K   C   K   M   E   L   D   I
KARAT   ATG ACT TAT GGT ATT TTG CTG GAT GGG TTG TGT GAC AAT GGC GAA CTA GAA GAG GCT TTG GGA ATA CTT GAT CAA ATG CAC AAG TGT AAG ATG GAA CTT GAT ATT

CMS     GGT ATA TAT AGT ATC ATC ATT CAC GGG TTG TGC AAT GCA AGT AAG ATC GAT GAT GCT TGG GAT CTA TTC TGT AGC CTC TCT CTC AAA GGA GTG AAG CGT GAT ATT
         G   I   Y   S   I   I   I   H   G   L   C   N   A   S   K   I   D   D   A   W   D   L   F   C   S   L   S   L   K   G   V   K   R   D   I
KARAT   GGT ATA TAT AGT ATC ATC ATT CAC GGG TTG TGC AAT GCA AGT AAG ATC GAT GAT GCT TGG GAT CTA TTC TGT AGC CTC TCT CTC AAA GGA GTG AAG CGT GAT ATT

CMS     CAG TCA TAC AAC ATA ATG TTG TCA GGA TTA TGT AAA AGG AGC TCA TTG TCT GAA GCG GAT GCA TTG TTT AGA AAA ATG AAG GAA GAT GGG TAT GAG CCA GAT GGT
         Q   S   Y   N   I   M   L   S   G   L   C   K   R   S   S   L   S   E   A   D   A   L   F   R   K   M   K   E   D   G   Y   E   P   D   G
KARAT   CAG TCA TAC AAC ATA ATG TTG TCA GGA TTA TGT AAA AGG AGC TCA TTG TCT GAA GCG GAT GCA TTG TTT AGA AAA ATG AAG GAA GAT GGG TAT GAG CCA GAT GGT

CMS      TGT ACG TAC AAT ACA CTT ATC AGA GCA CAT CTT CGA GGT AGT GAC ATA ACA ACT TCA GTT CAA CTC ATT GAA GAA ATG AAG AGG TGT GGG TTC TCT TCA GAT GCT
          C   T   Y   N   T   L   I   R   A   H   L   R   G   S   D   I   T   T   S   V   Q   L   I   E   E   M   K   R   C   G   F   S   S   D   A
KARAT    TGT ACG TAC AAT ACA CTT ATC AGA GCA CAT CTT CGA GGT AGT GAC ATA ACA ACT TCA GTT CAA CTC ATT GAA GAA ATG AAG AGG TGT GGG TTC TCT TCA GAT GCT

CMS      TCC ACC GTA AAG ATT GTT ATG GAT ATG CTA TCA AGT GGT
          S   T   V   K   I   V   M   D   M   L   S   S   G
KARAT    TCC ACC GTA AAG ATT GTT ATG GAT ATG CTA TCA AGT GGT

CMS     GAA TTG GAC AAA AGC TTT CTA AAT ATG CTT TCT GGT CCT TTT GGA GAC AAA TCA TCA TTG
         E   L   D   K   S   F   L   N   M   L   S   G   P   F   G   D   K   S   S   L
KARAT   GAA TTG GAC AAA AGC TTT CTA AAT ATG CTT TCT GGT CCT TTT GGA GAC AAA TCA TCA TTG

CMS     TTG GAT TGA
         L   D   -
KARAT   TTG GAT TGA
```
